# A multi‐faceted approach testing the effects of previous bacterial exposure on resistance and tolerance

**DOI:** 10.1111/1365-2656.12953

**Published:** 2019-03-06

**Authors:** Megan A. M. Kutzer, Joachim Kurtz, Sophie A. O. Armitage

**Affiliations:** ^1^ Institute for Evolution and Biodiversity University of Münster Münster Germany; ^2^ IST Austria Klosterneuburg Austria; ^3^ Institute of Biology Freie Universität Berlin Berlin Germany

**Keywords:** *Drosophila* Genetic Reference Panel, *Drosophila melanogaster*, ecological immunology, immune memory, immune priming, persistent infection, resistance, tolerance

## Abstract

1. Hosts can alter their strategy towards pathogens during their lifetime; that is, they can show phenotypic plasticity in immunity or life history. Immune priming is one such example, where a previous encounter with a pathogen confers enhanced protection upon secondary challenge, resulting in reduced pathogen load (i.e., resistance) and improved host survival. However, an initial encounter might also enhance tolerance, particularly to less virulent opportunistic pathogens that establish persistent infections. In this scenario, individuals are better able to reduce the negative fecundity consequences that result from a high pathogen burden. Finally, previous exposure may also lead to life‐history adjustments, such as terminal investment into reproduction.
2. Using different *Drosophila melanogaster* host genotypes and two bacterial pathogens, *Lactococcus lactis* and *Pseudomonas entomophila*, we tested whether previous exposure results in resistance or tolerance and whether it modifies immune gene expression during an acute‐phase infection (one day post‐challenge). We then asked whether previous pathogen exposure affects chronic‐phase pathogen persistence and longer‐term survival (28 days post‐challenge).
3. We predicted that previous exposure would increase host resistance to an early stage bacterial infection while it might come at a cost to host fecundity tolerance. We reasoned that resistance would be due in part to stronger immune gene expression after challenge. We expected that previous exposure would improve long‐term survival, that it would reduce infection persistence, and we expected to find genetic variation in these responses.
4. We found that previous exposure to *P. entomophila* weakened host resistance to a second infection independent of genotype and had no effect on immune gene expression. Fecundity tolerance showed genotypic variation but was not influenced by previous exposure. However, *L. lactis* persisted as a chronic infection, whereas survivors cleared the more pathogenic *P. entomophila* infection.
5. To our knowledge, this is the first study that addresses host tolerance to bacteria in relation to previous exposure, taking a multi‐faceted approach to address the topic. Our results suggest that previous exposure comes with transient costs to resistance during the early stage of infection in this host–pathogen system and that infection persistence may be bacterium‐specific.

## 1 INTRODUCTION

Pathogens are ubiquitous and represent a driving evolutionary force, in response to which hosts have evolved defence strategies such as resistance and tolerance (Råberg, Graham, & Read, 2009; Roy & Kirchner, 2000). If a pathogen successfully circumvents a host's barrier defences, the host can resist the infection by clearing the pathogen or by targeting pathogen replication rate (Best, White, & Boots, 2008). However, the immune system can be costly in terms of resource use or self‐damage (Khan, Agashe, & Rolff, 2017; Sadd & Siva‐Jothy, 2006) and the pathogen itself can directly damage the host. In response to these factors, hosts can evolve tolerance, which limits the negative fitness or health effects associated with a given pathogen load, without directly targeting pathogen numbers (Råberg et al., 2009). These defence strategies can be both plastic and innate, in that their expression is shaped by a combination of the external host environment, host genetic factors and the pathogen itself (Ayres & Schneider, 2008; Graham et al., 2011; Howick & Lazzaro, 2014; Kutzer & Armitage, 2016b; Råberg et al., 2009). Therefore, we suggest that it is likely that resistance and tolerance can be shaped by a host's previous experience with a pathogen.

Previous (or primary) pathogen exposure can result in immunological memory, where individuals have enhanced protection upon a secondary exposure (hereafter termed challenge) to the same pathogen. Evidence for this phenomenon is found across the animal kingdom (Milutinović & Kurtz, 2016; Pradeu & Du Pasquier, 2018), with multiple examples coming from invertebrate taxa (Dhinaut, Chogne, & Moret, 2018; Kurtz & Franz, 2003; Moret & Siva‐Jothy, 2003; Pham, Dionne, Shirasu‐Hiza, & Schneider, 2007; Pinaud et al., 2016; Roth, Sadd, Schmid‐Hempel, & Kurtz, 2009; Sadd & Schmid‐Hempel, 2006). Here, the phenomenon is often referred to as immune priming (Little & Kraaijeveld, 2004). The effects of priming can be broad, ranging from coarse specificity (e.g., Boman, Nilsson, & Rasmuson, 1972; Moret & Siva‐Jothy, 2003) to highly specific responses that differentiate at the level of bacterial strain (Futo, Sell, Kutzer, & Kurtz, 2017; Roth et al., 2009). *Drosophila melanogaster*, the model used in this study, shows some evidence of priming (Boman et al., 1972; Pham et al., 2007), although not towards all tested pathogens (Longdon, Cao, Martinez, & Jiggins, 2013; Pham et al., 2007). In general, previous pathogen exposure does not always lead to immune priming. Some studies have found no support (González‐Tokman, González‐Santoyo, Lanz‐Mendoza, & Córdoba Aguilar, 2010; Reber & Chapuisat, 2012), while others found protection with specific pathogens (Roth et al., 2009).

Many empirical priming studies use survival readouts as evidence for increased protection. Of 37 insect and crustacean priming studies reviewed by Contreras‐Garduño et al. (2016), around 50% assayed survival to understand if previous pathogen exposure confers increased protection. Priming is thought to act by enhancing host resistance, yet pathogen load after challenge is rarely assayed (but see Boman et al., 1972; Sadd & Schmid‐Hempel, 2006; Pham et al., 2007). Furthermore, infections can persist in the insect haemocoel for weeks (Haine, Moret, Siva‐Jothy, & Rolff, 2008), and although previous exposure can result in dramatic declines in bacteria load after a challenge (Boman et al., 1972; Pham et al., 2007), and even eliminate the pathogen in some hosts (Pham et al., 2007), this is not always the case (e.g., Contreras‐Garduño, Rodriguez, Rodriguez, Alvarado‐Delgado, & Lanz‐Mendoza, 2014; Rodrigues, Brayner, Alves, Dixit, & Barillas‐Mury, 2010). The question therefore arises as to whether previous exposure affects pathogen persistence in the longer term.

Given theoretical and empirical work suggesting the costs of immune defence (e.g., Armitage, Thompson, Rolff, & Siva‐Jothy, 2003; Boots & Begon, 1993; Kraaijeveld & Godfray, 1997) and priming in particular (Best, Tidbury, White, & Boots, 2013; Tate & Rudolf, 2012), we might expect that increased survival after previous exposure comes with costs. For example, this might manifest itself through life‐history trade‐offs and result in lower host fecundity. Darwinian fitness is comprised of survivorship and reproductive rate. Despite this, reproductive output has rarely been examined empirically in a priming context (but see Contreras‐Garduño et al., 2014). Indeed, trade‐offs or terminal fecundity investment (Adamo, 1999) might explain why survival differences are not seen after previous pathogen exposure in some cases.

We and others have demonstrated that the expression of host defence strategies is context‐dependent (reviewed in Kutzer & Armitage, 2016a). That is, resistance and tolerance will vary as a function of host environmental fluctuations, host genotype and immunopathology (Howick & Lazzaro, 2014; Jackson et al., 2014; Kutzer & Armitage, 2016b; Råberg, Sim, & Read, 2007; Sternberg et al., 2012). Given that immune priming also seems to be context‐dependent and plastic, we asked whether priming in *D. melanogaster* acts through resistance or tolerance. Theoretical work suggests that priming can be both resistance‐ and tolerance‐mediated, with tolerance‐based systems being analogous to leaky vaccinations in vertebrate systems (Gandon, Mackinnon, Nee, & Read, 2001; Read et al., 2015; Tate, 2017). Therefore, using different *D. melanogaster* genotypes as our hosts and two bacterial species as our pathogens, we endeavoured to take a multi‐angled perspective on the effects of previous pathogen exposure, addressing how it modulates acute‐phase post‐challenge resistance, fecundity tolerance and immune gene expression, and whether previous exposure affects chronic‐phase pathogen persistence and longer‐term survival. In a first experiment addressing the acute phase after challenge, here defined as 24 hrs post‐challenge, we predicted that: (a) previous pathogen exposure will increase resistance (lower bacteria load) after challenge compared to unprimed individuals; (b) given life‐history trade‐offs, if priming is costly, increased resistance may come at a cost to fecundity tolerance, so that individuals previously exposed to pathogens will have a greater reduction in fitness for a given pathogen load than individuals initially receiving a control injection; and (c) flies with prior pathogen experience will have stronger immune gene expression after challenge than those without such experience. In a second experiment testing the chronic phase after challenge, here defined as 28 days, we predicted that: (d) survival would be higher in flies previously exposed to bacteria; (e) our bacteria species may form persistent infections, and if so, previous exposure would affect infection persistence; and lastly, (f) given that some host genotypes are more likely to become primed than others (Khan, Prakash, & Agashe, 2016; Portela et al., 2013), we predicted that the responses in the aforementioned predictions (a–e) may show genetic variation.

## 2 MATERIALS AND METHODS

### 2.1 *Drosophila melanogaster* culture conditions

**Figure 1:**
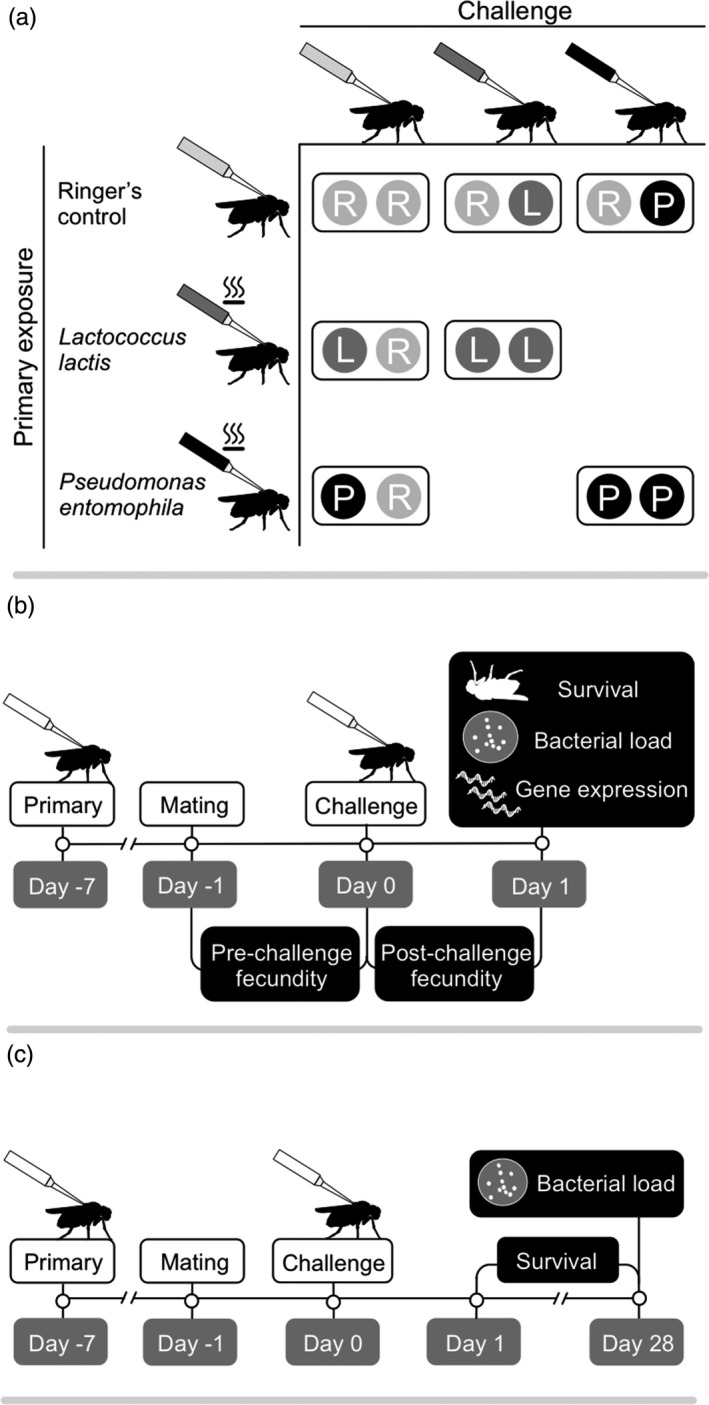
Experimental designs. (a) The primary exposure (also termed previous exposure) and challenge treatment combinations used for both experiments 1 and 2. Bacteria used for the primary exposure were heat‐killed, whereas those used for challenge were alive. (b) The timelines for experiment 1 and (c) experiment 2

In experiment 1, we used four fly lines (RAL350, RAL367, RAL379 and RAL765) from the *D. melanogaster* genetic reference panel (DGRP; Mackay et al., 2012). In experiment 2, we used two lines (RAL379 and RAL765). For an overview of the experimental designs, see Figure 1. The lines were selected based on the results of a previous experiment (Kutzer, Kurtz, & Armitage, 2018a), where we found that each varied in fecundity and in their capacity to resist bacteria. The stocks were maintained by placing adults onto new food every 2 weeks. All lines were maintained at 25°C and 70% relative humidity on a 12:12‐hrs light–dark cycle, and reared on a standard sugar yeast agar medium (1.5% agar, 5% sugar, 10% brewer's yeast, 3% nipagin, 0.3% propionic acid; SYA medium, Bass et al., 2007). For experiment 1, the procedures described below were repeated six times to produce six experimental replicates, and for experiment 2, they were repeated three times. Treatments within experiments were performed in randomized blocks.

### 2.2 Experimental animals

We raised the individuals used in experiment 1, as well as their parents, at constant larval densities as described in Kutzer and Armitage (2016b) with the following modifications. Between 300 and 500 individuals from each of the four DGRP lines were placed in embryo collection cages to generate the F1 generation for each replicate. We collected 300–400 larvae for each genotype for both the F1 and F2 generations per replicate following Kutzer et al. (2018a). When the F2 generation (i.e., the experimental animals) had eclosed, we allocated 120 virgin females of each genotype into separate vials in groups of 10 and 120 virgin males of each genotype were allocated to vials in groups of 20. Individuals for experiment 2 were raised at constant larval density, but not their parents. We collected 1,100 larvae per replicate for each genotype. Once the adults had eclosed, using CO_2_ anaesthetization, 300 virgin females of each genotype were allocated to separate vials in groups of 10 and 300 virgin males of each genotype in groups of 20.

### 2.3 Preparation of bacteria for heat killing and primary injections

We used *Lactococcus lactis*, a Gram‐positive bacterium, and *Pseudomonas entomophila*, a Gram‐negative bacterium. These species can be considered as opportunistic pathogens. The *L. lactis* strain was isolated from a wild‐caught *D. melanogaster* in State College, Pennsylvania (Lazzaro, 2002), and the *P. entomophila* strain was isolated from a wild‐caught *D. melanogaster* in Guadeloupe (Vodovar et al., 2005). Both bacteria have different pathogenicities. *P. entomophila* causes earlier host death when injected at the same dose as *L. lactis*. Aliquots of *L. lactis* and *P. entomophila* were stored in 34.4% glycerol at −80°C. We plated out *L. lactis* (gift from Brian Lazzaro) on lysogeny broth (LB) agar and plated out *P. entomophila* (gift from Bruno Lemaitre) on LB agar containing 1% milk to select for protease positive clones (Neyen, Poidevin, Roussel, & Lemaitre, 2012) and then incubated the plates for approximately 24 hrs at 30°C. We inoculated 100 ml of sterile LB with four clones of each bacteria species into 500‐ml Erlenmeyer flasks and left the bacteria to grow overnight at 30°C, at 200 rpm. The next morning, we centrifuged the cultures at 2,880 rcf at 4°C for 15 min and then removed the supernatant. The bacteria were washed twice in sterile *Drosophila* Ringer's solution (Werner et al., 2000), heat‐killed at 90°C in a heating block, counted using a Thoma counting chamber, diluted in Ringer's solution to 1 × 10^8^ cells/ml (Roth et al., 2009) and then frozen at −80°C in 1,000 μl aliquots for later use. The heat‐killed bacterial solutions were plated out on LB agar and incubated overnight at 30°C to confirm that no live bacteria were present.

When the virgin females were approximately 4 days old, they were anaesthetized in groups of 10 with light CO_2_ in the early afternoon and then injected on the right lateral side of the thorax with 18.4 nl of either heat‐killed *P. entomophila*, heat‐killed *L. lactis,* or sterile Ringer's solution (R) that had been frozen at −80°C, using a Nanoject II (Drummond Scientific). These primary exposure injections yielded a dose of approximately 1,840 heat‐killed bacteria per fly. We plated out each heat‐killed bacterial aliquot after injection to check for contamination and to ensure that we had not introduced any live bacteria into the flies. We checked survival 6 days after injection on the day of mating for each replicate. The survival of each of the six replicates between the primary exposure and mating was unaffected by primary exposure treatment, but was affected by genotype in experiment 1 (GLMM, *χ* ^2^ **=* *25.21, *p *<* *0.0001; RAL350: 88.4% survived, *n *=* *599, RAL367: 94.2%, *n *=* *583, RAL379: 95.6%, *n *=* *590, RAL765: 91.2%, *n *=* *580). There was no effect of genotype or primary exposure on survival in experiment 2. We chose the age of the flies and the timing between the primary exposure and the challenge based on Pham et al. (2007).

### 2.4 Mating assay

To ensure that all females were mated once, we observed individual matings 6 days after primary exposure (Supporting Information Appendix S1). Mated females were chosen at random for infections the following day (experiment 1: replicates 1–4: *n *=* *146–166 females per replicate, replicates 5 and 6: 168 females per replicate + 48 extra for qPCR per replicate, for a total of 959 animals for the resistance/tolerance analysis; experiment 2: replicates 1–3: *n *=* *252 females per replicate, for a total of 756 females).

### 2.5 Bacterial preparation and challenge injections

Seven days after primary exposure, *L. lactis* and *P. entomophila* were prepared for infections with live bacteria (“challenge” injections) following Kutzer and Armitage (2016b), with the modification that females were injected in the lateral left side of the thorax. Each female was injected with either 18.4 nl of Ringer's solution, live *L. lactis* (approximately 1,840 bacteria per fly) or live *P. entomophila* (approximately 92 bacteria per fly). This resulted in seven primary exposure‐challenge combinations (Figure 1a): Ringer's–Ringer's (R−R), Ringer's–live *L. lactis* (R−L), Ringer's–live *P. entomophila* (R−P), heat‐killed *L. lactis*–Ringer's (L−R), heat‐killed *P. entomophila*–Ringer's (P−R), heat‐killed *L. lactis*–live *L. lactis* (L−L) and heat‐killed *P. entomophila*–live *P. entomophila* (P−P). Females were returned to 25°C and 70% relative humidity after injection. We diluted the leftover injection bacterial aliquots to 1 × 10^3^ cells/ml and plated 50 μl of each onto LB plates, which should have yielded 50 CFUs. Bacterial counts from each aliquot ranged from 33 to 86 (experiment 1) and 26 to 78 (experiment 2) CFUs for *L. lactis* and 14 to 62 (experiment 1) and 21 to 43 (experiment 2) for *P. entomophila*. No bacteria grew in the Ringer's aliquots, and we found no evidence of contamination for any bacterial replicate.

### 2.6 Experiment 1

#### 2.6.1 Fecundity measure and fecundity tolerance

For a graphical overview, see Figure 1b. Pre‐challenge fecundity was measured as the total number of adult offspring produced by females in the ~26 hrs between mating and challenge injections, and post‐challenge fecundity was measured as the total number of adult offspring produced by each female in the 24 hrs post‐challenge (hereafter termed one day post‐challenge, DPC). Once we had removed the females from their vials for either the bacterial load assay or gene expression assay (see below), the vials were kept at 25°C and 70% relative humidity, for 12 days to allow the offspring to complete their development. The vials were frozen to allow us to count adult offspring at a later date. Range tolerance (Little, Shuker, Colegrave, Day, & Graham, 2010) was assessed as the relationship (slope) between fly fecundity and individual‐level bacterial load (see below). Unlike point tolerance, range tolerance considers fecundity over a range of parasite loads, which result from naturally occurring variation in responses in our system because each animal received the same inoculation dose (Kutzer & Armitage, 2016a). A steeper negative slope indicates a greater fitness loss for an increase in pathogen load, that is lower tolerance.

#### 2.6.2 Bacteria load assay one DPC

Previous work demonstrated that bacteria load declines after 24 hrs so we reasoned that this is where we might see the greatest differences in the bacterial loads of previously exposed and control flies (Kutzer & Armitage, 2016b). We assayed bacterial load at one DPC by plating dilutions of whole fly homogenates onto LB agar plates to quantify host resistance (the inverse of bacterial load) according to Kutzer and Armitage (2016b). Colony morphology and colour were consistent with the injected bacteria in all cases (e.g., see methods of Lazzaro, Sackton, & Clark, 2006).

#### 2.6.3 RNA extraction and RT‐qPCR one DPC

To test whether previous exposure affected the strength of the immune response post‐challenge, for treatments involving *L. lactis* and its controls, we took a candidate gene approach and assayed the expression of two antimicrobial peptides, *Drosomycin* (*Drs*) and *Metchnikowin* (*Mtk*), both of which are activated after infection with Gram‐positive bacteria (e.g., *Drs*: Leulier et al. 2000; *Mtk*: Levashina et al. 1995; Linder et al. 2008; Leulier et al. 2000). We also assayed the expression of a somatically diversified cell surface immune receptor, *Down syndrome cell adhesion molecule 1* (*Dscam1*) (Watson et al., 2005). A subset of flies from replicates 5 and 6 were individually placed into 1.5‐ml microcentrifuge tubes and frozen in liquid nitrogen and stored at −80°C for gene expression analyses, instead of being homogenized to assay bacterial load. We froze flies from all four genotypes and the following treatments: L−L, L−R, R−L, and R−R. RNA extraction, DNase treatment, reverse transcription and qPCRs were performed as described in Supporting Information Appendix S1.

### 2.7 Experiment 2

#### 2.7.1 Survival and bacterial load assay 28 DPC

For a graphical overview, see Figure 1c. After challenge, females were kept at 25°C and 70% relative humidity, and placed into fresh food vials every 7 days. Fly survival was censused daily until 28 DPC. We assayed bacterial load at 28 DPC using the same methods as described for one DPC, except that we also homogenized Ringer's‐challenged flies. Fly mortality meant that we could only assay a small subset of survivors for their bacterial load. The sample sizes for each of the treatments and genotypes were as follows, where the numbers in parentheses are the number of flies for the two genotypes (RAL379 and RAL765): L–L (9/5); L–R (11/11); P–P (1/10); P–R (11/11); R–L (10/5); R–P (1/9); R–R (11/11).

### 2.8 Statistical analyses

Statistical analyses were performed in R version 3.3.3 (R Core Team, 2016). For all models, see Supporting Information Appendix S1. We used experimental replicate and block nested within replicate as random effects (random intercept) and fly identity to control for overdispersion where necessary to control for variance among replicates or an unbalanced design due to missing flies. Figures were plotted with ggplot2 (Wickham, 2009) and Microsoft Excel.

## 3 RESULTS

### 3.1 Experiment 1: Survival and resistance one DPC

**Figure 2:**
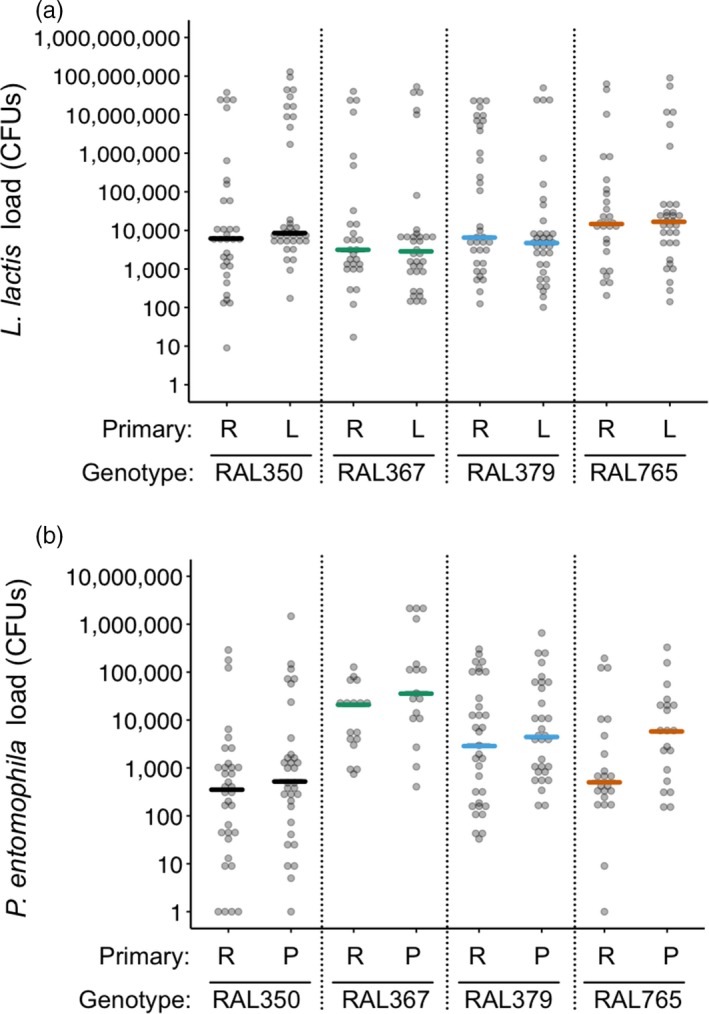
Bacteria load one day post‐challenge (DPC). Bacteria load on the *y*‐axis was quantified as the number of colony‐forming units (CFUs) per fly. The analyses were performed using *ln(x)*‐transformed data, but for ease of interpretation, we present untransformed values on a *log(x)* scale. The *x*‐axis shows fly genotypes and the primary exposure treatments, where R: Ringer's, L: *Lactococcus lactis* and P: *Pseudomonas entomophila*. (a) There was no effect of the primary exposure on *L. lactis* load post‐challenge. (b) Flies injected with heat‐killed *P. entomophila* had higher *P. entomophila* loads than the Ringer's‐injected flies. Coloured lines show median bacterial loads. For statistics, see Table 1

Survival in the challenge controls one DPC was high and unaffected by genotype or previous pathogen exposure (Supporting Information Figure S1A). Previous exposure to heat‐killed *L. lactis* had no effect on survival after challenge with *L. lactis* (Supporting Information Table S2) but survival differed among genotypes in the *L. lactis*‐challenged groups (Supporting Information Table S2; Figure S1B). Previous exposure to heat‐killed *P. entomophila* also did not confer a survival advantage after a secondary challenge, but survival differed among genotypes (Supporting Information Table S2; Figure S1C). We observed no effect of previous exposure or genotype on resistance in flies that had been challenged with *L. lactis* (Table 1; Figure 2a). However, a primary injection with heat‐killed *P. entomophila* decreased resistance (increased bacteria load) after live challenge with *P. entomophila*, and resistance also differed among genotypes (Table 1; Figure 2b). There was no relationship between mean bacteria load for each of the bacteria (Supporting Information Figure S2A).

**Table 1:** The effect of genotype and primary exposure treatment on the response variable bacterial load one day post‐challenge (DPC)

Tested effect	Model 2a: *L. lactis*				Model 2b: *P. entomophila*			
	*F*	*df*	Resid. *df*	*p*	*F*	*df*	Resid. *df*	*p*
Genotype	1.65	3	239.26	0.18	19.39	3	187.02	<**0.0001**
Primary	0.05	1	240.67	0.83	10.87	1	184.78	**0.001**
Genotype × Primary	2.29	3	239.37	0.08	0.51	3	187.52	0.68

L. lactis: Lactococcus lactis; P. entomophila: Pseudomonas entomophila. For model 2a, primary refers to injection with Ringer's or heat‐killed *L. lactis*, and for model 2b, it refers to Ringer's or heat‐killed *P. entomophila*.

Values in bold are statistically significant.

### 3.2 Experiment 1: Fecundity and fecundity tolerance one DPC

**Figure 3:**
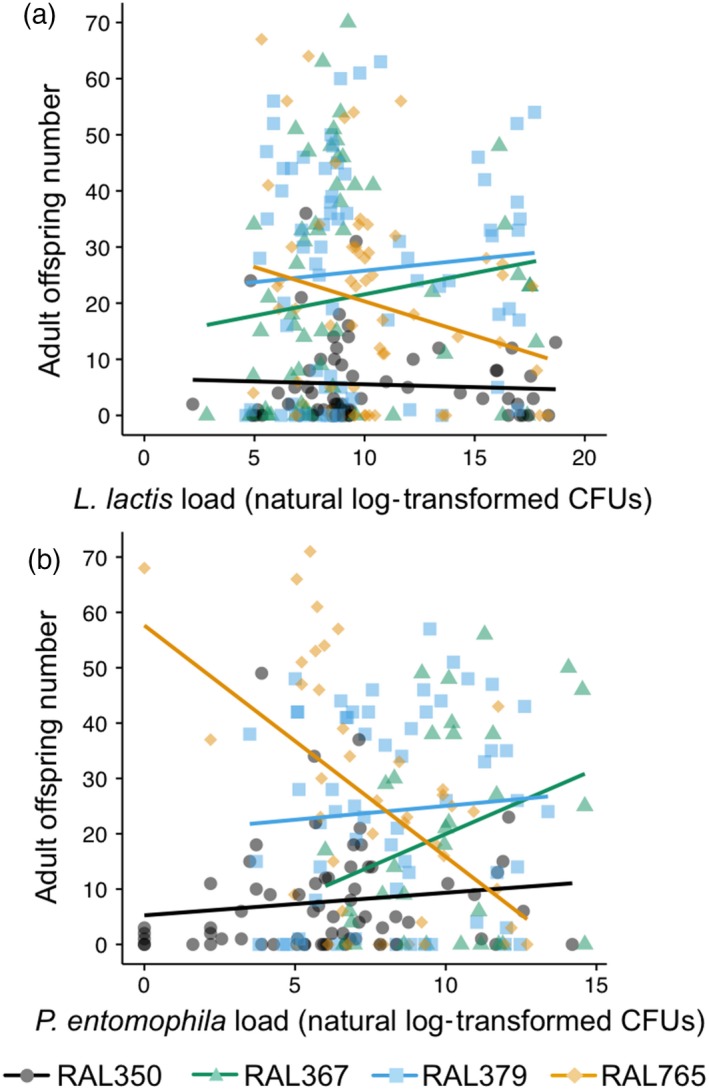
Fecundity tolerance varies according to genotype one day post‐challenge (DPC). Tolerance reaction norms are plotted for each genotype infected with (a) *Lactococcus  lactis* and (b) * Pseudomonas  entomophila*. The natural log of bacterial load (CFU) is plotted against fecundity one DPC. Each data point represents bacterial load and fitness for one fly. For statistics, see Table 2

Post‐challenge fecundity differed among genotypes and was correlated with pre‐challenge fecundity (Supporting Information Table S3). However, post‐challenge fecundity was unaffected by challenge or primary injection with either *L. lactis* or *P. entomophila* (Supporting Information Table S3; Figure S3A–C). Previous exposure did not affect fecundity tolerance towards *L. lactis* or *P. entomophila* (Table 2, lack of significant interactions between CFU × Priming; Supporting Information Figure S4). However, genotypes differed in their tolerance towards *L. lactis* and *P. entomophila* challenge (significant interactions between CFU × Genotype in Table 2); for example, in response to both bacterial infections RAL765 showed a reduction in offspring production with an increase in bacterial load (negative tolerance slope), contrasting with RAL367, which had positive tolerance slopes (Figure 3). Fecundity was positively correlated across bacteria species (Supporting Information Figure S2B), which might be expected given that bacterial infection did not affect fecundity in either case, but there was no significant relationship between fecundity and *L. lactis* load (Supporting Information Figure S2C), or between tolerance towards the two pathogens (Supporting Information Figure S2D).

**Table 2:** The effects of bacteria load (CFU), genotype and primary exposure treatment (Ringer's or heat‐killed *Lactococcus lactis* for model 4a; Ringer's or heat‐killed *Pseudomonas entomophila* for model 4b) on the response variable fecundity tolerance one day post‐challenge (DPC)

Tested effect	Model 4a: *L. lactis*			Model 4b: *P. entomophila*		
	χ^2^	*df*	*p*	χ^2^	*df*	*p*
CFU	0.41	1	0.52	0.39	1	0.53
Genotype	41.54	3	<**0.0001**	22.78	3	<**0.0001**
Primary	0.07	1	0.78	0.0009	1	0.98
CFU × Genotype	8.85	3	**0.03**	9.75	3	**0.02**
CFU × Primary	0.26	1	0.61	2.88	1	0.09
Genotype × Primary	2.73	3	0.43	1.11	3	0.77
CFU × Genotype × Primary	3.33	3	0.34	4.71	3	0.19

Significant interactions between CFU and genotype indicate genetic variation in fecundity tolerance.

Values in bold are statistically significant.

### 3.3 *Experiment 1: Gene expression in L. lactis*‐*injected flies one DPC*

**Figure 4:**
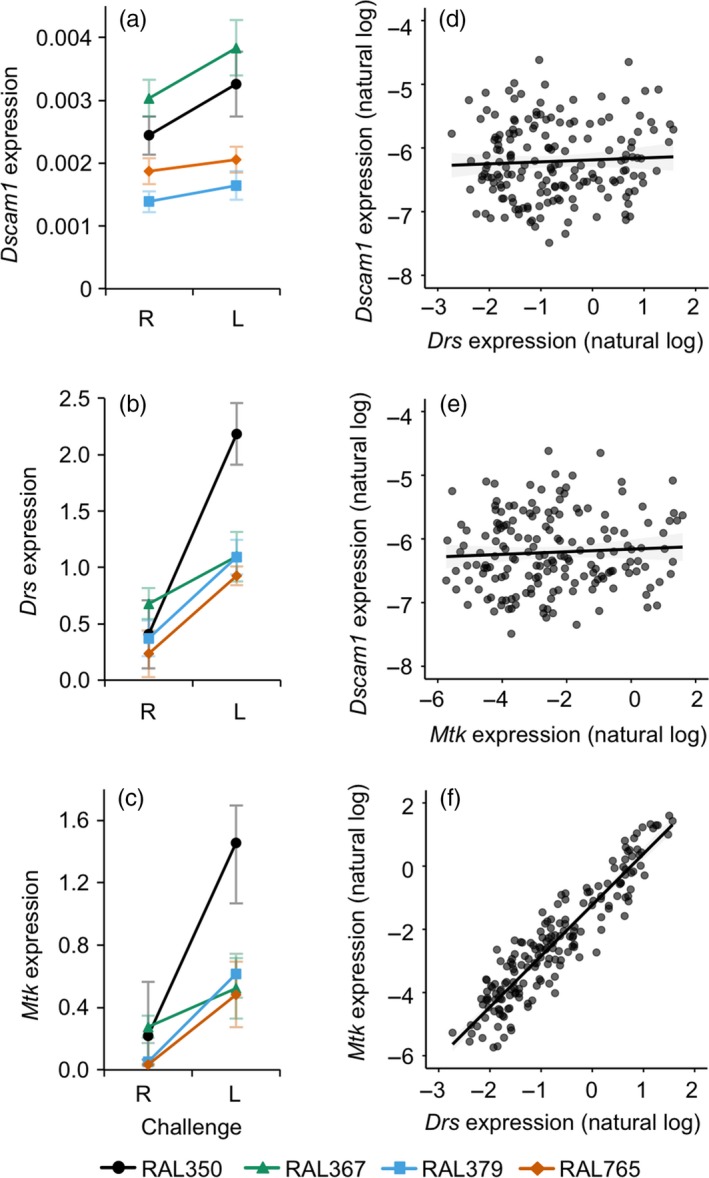
Target gene expression relative to reference gene expression one day post‐challenge (DPC). R indicates Ringer's, and L indicates *Lactococcus lactis*. The left panel shows gene expression (fold expression values, 2^‐ΔCT^) according to challenge and genotype, and the right panel shows correlations between the expression level of different gene pairs. (a) *Dscam1*, where expression increased significantly upon challenge; (b) *Drosomycin* and (c) *Metchnikowin* where the interactions between genotype and challenge in both panels are statistically significant. Symbols and error bars indicate means with standard errors, where *n *=* *16‐24 per symbol. Reaction norms are plotted for each genotype. The legend at the bottom indicates the genotypes in panels a–c. For statistics, see Table 3. (d–f) Correlations between the expression of the three genes. Each data point is from one individual; the lines illustrate linear fits. For statistics, see Results in text

None of the genes varied their expression according to previous pathogen exposure, but all three genes were significantly up‐regulated in response to a live bacterial challenge and varied according to genotype (Table 3, Figure 4a–c). Challenge affected *Drosomycin* and *Metchnikowin* expression in a genotype‐dependent way (Table 3), where RAL350 had a stronger increase in expression after infection compared to the other three genotypes. *Dscam1* expression did not correlate with the expression of either *Drs* (*χ* ^2^ = 0.494, *df *=* *1, *p *=* *0.482; Figure 4d) or *Mtk* (*χ* ^2^ **=* *0.646, *df *=* *1, *p *=* *0.422; Figure 4e), but there was a strong positive relationship between *Drs* and *Mtk* expression (*χ* ^2^ **=* *1,097.3, *df *=* *1, *p *<* *0.0001; Figure 4f). There were no significant correlations between mean or median gene expression and bacterial load (Supporting Information Figure S5).

**Table 3:** The effects of genotype, primary exposure treatment (R or L) and challenge (R or L) on the response variables *Dscam1*,* Drosomycin* and *Metchnikowin* gene expression one day post‐challenge (DPC)

Tested effect	Model 5a. *Dscam1*				Model 5b. *Drosomycin*				Model 5c. *Metchnikowin*			
	*df*	χ^2^	*p*	*p* ^BH^	*df*	χ^2^	*p*	*p* ^BH^	*df*	χ^2^	*p*	*p* ^BH^
Genotype	3	71.54	<**0.0001**	**0.00015**	3	16.48	**0.0009**	**0.0009**	3	22.63	<**0.0001**	**0.00015**
Primary	1	0.0028	0.96		1	0.99	0.32		1	0.10	0.75	
Challenge	1	5.51	**0.019**	**0.019**	1	95.78	<**0.0001**	**0.00015**	1	99.72	<**0.0001**	**0.00015**
Genotype × Primary	3	3.06	0.38	0.38	3	8.04	**0.045**	0.135	3	3.52	0.32	0.38
Genotype × Challenge	3	1.03	0.79	0.79	3	18.62	**0.0003**	**0.0009**	3	13.88	**0.003**	**0.0045**
Primary × Challenge	1	3.19	0.074		1	2.13	0.14		1	2.46	0.12	
Genotype × Primary × Challenge	3	1.21	0.75		3	3.72	0.29		3	0.56	0.90	

*p* ^BH^ indicates the *p*‐values after adjustment for multiple testing using the Benjamini–Hochberg correction. We only present the corrected values for terms that had a significant effect on at least one gene.

Values in bold are statistically significant.

### 3.4 Experiment 2: Survival and resistance 28 DPC

**Figure 5:**
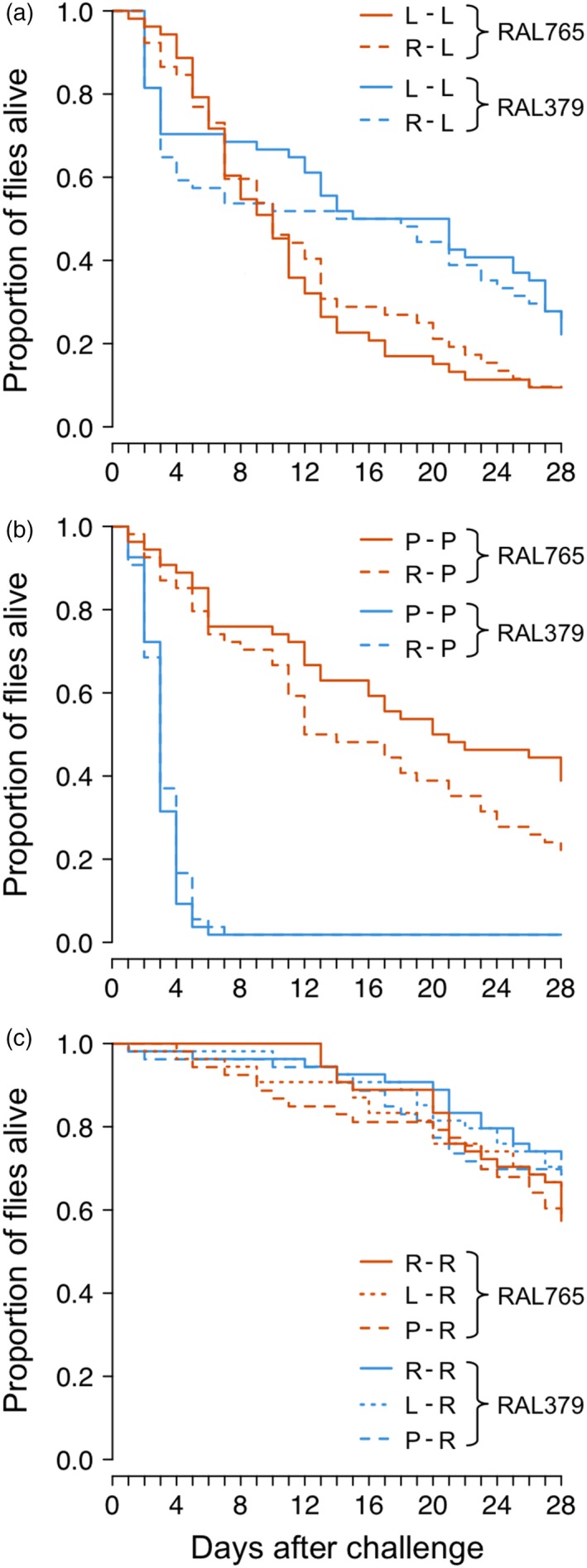
Effect of primary exposure and genotype on survival to 28 day post‐challenge (DPC). Flies were challenged with (a) *Lactococcus  lactis* (*L*) (b) *Pseudomonas entomophila* (P) or (c) Ringer's (R). Genotype survival differed significantly when flies were infected with bacteria. Legends indicate the genotypes and treatments. Each line is the cumulative survival of between 52 and 54 individuals. For statistics, see Table 4

Genotype was the only factor to significantly affect survival over the 28 days following challenge with *L. lactis* or *P. entomophila* (Figure 5; Table 4): RAL765 had lower survival when challenged with *L. lactis*, but a higher survival when challenged with *P. entomophila* relative to RAL379. There was no significant effect of primary exposure treatment on survival for either of the genotypes. The survival of Ringer's‐challenged flies was not affected by primary exposure treatment or genotype.

**Table 4:** The effects of genotype and primary exposure treatment on the response variable survival 28 day post‐challenge (DPC)

Tested effect	Model 6a Ringer's			Model 6b *L. lactis*			Model 6c *P. entomophila*		
	*df*	χ^2^	*p*	*df*	χ^2^	*p*	*df*	χ^2^	*p*
Genotype	1	3.19	0.074	1	8.42	**0.0037**	1	124.1	<**0.0001**
Primary	1	0.81	0.67	1	0.059	0.81	1	0.60	0.44
Genotype × Primary	1	0.23	0.90	1	0.49	0.48	1	2.48	0.12

L. lactis: Lactococcus lactis; P. entomophila: Pseudomonas entomophila. For model 6a, previous exposure has three levels (L, P & R) and two levels for models 6b (L & R) and 6c (P & R).

Values in bold are statistically significant.

**Figure 6:**
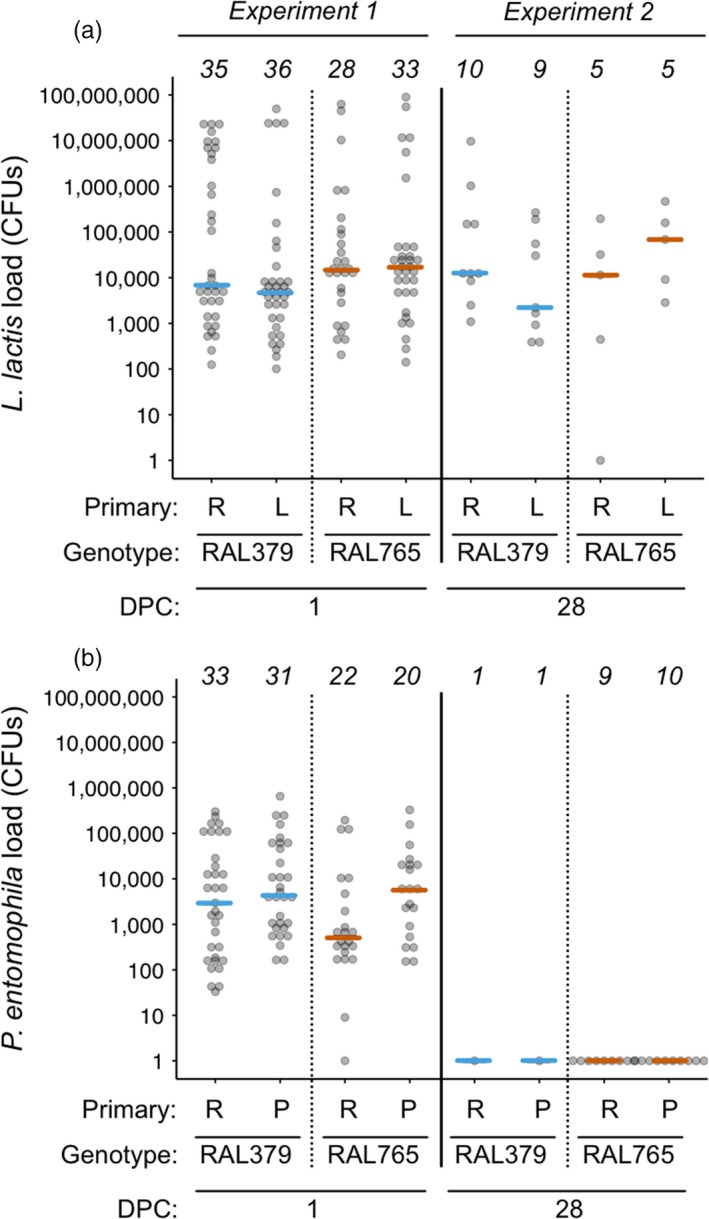
Bacteria load one and 28 day post‐challenge (DPC). (a) *Lactococcus lactis* and (b) *Pseudomonas entomophila* load quantified as the number of colony‐forming units (CFUs) per individual fly. The analyses were performed using ln(*x*)‐transformed data, but for ease of interpretation, we here present untransformed values on a log(*x*) scale. Sample sizes are given across the tops of the figures. In experiment 1, flies were homogenized at one DPC, and in experiment 2 at 28 DPC. Each dot shows the load for one fly, and the coloured lines indicate medians. For statistics, see Table 5

*Lactococcus lactis* load 28 DPC (mean ± 1 *SE*: 4.3 × 10^5^ ± 3.3 × 10^5^) was on average one order of magnitude lower than one DPC (4.2 × 10^6^ ± 1.1 × 10^6^), yet still approximately 200 times higher than the challenge dose. *L. lactis* load was unaffected by previous exposure and/or genotype (Table 5, Figure 6). All flies that had been challenged with live *P. entomophila* had cleared the infection by 28 DPC (or the level was below the detection limit), although it is important to note that only two RAL379 flies survived the infection (Figure 6). No bacteria grew from homogenates of the Ringer's‐challenged flies.

**Table 5:** The effect of genotype and primary exposure treatment (R or L) on the response variable *Lactococcus lactis* load 28 day post‐challenge (DPC) (model 7)

Tested effect	*F*	*df*	Resid. *df*	*p*
Genotype	0.058	1	22.31	0.81
Primary	0.061	1	24.46	0.81
Genotype × Primary	3.22	1	23.92	0.085

## 4 DISCUSSION

Our goal was to provide a multi‐angled perspective on acute and chronic effects of previous pathogen exposure on resistance and tolerance. Contrary to our expectations, individuals previously exposed to *P. entomophila* tended to have higher bacterial loads than previously non‐exposed individuals regardless of genotype, suggesting that a primary injection with *P. entomophila* may later be costly. Fecundity tolerance was not affected by previous pathogen experience, but varied by genotype in response to challenge with both pathogens. Antimicrobial peptide gene expression also varied according to host genotype and challenge. Long‐term survival was solely genotype‐dependent. Each genotype's survival trajectory diverged after bacterial infection. Furthermore, the more pathogenic bacterium, *P. entomophila*, was cleared by the surviving individuals, or was below the detection limit, whereas *L. lactis* persisted until at least 4 weeks post‐challenge despite a clear cost of infection.

### 4.1 Previous pathogen exposure can decrease infection resistance

Previous pathogen exposure is predicted to confer resistance to the same pathogen. The previous immune priming studies that quantified pathogen load found that priming enhanced host resistance (Contreras‐Garduño et al., 2014; Pham et al., 2007; Rodrigues et al., 2010; Sadd & Schmid‐Hempel, 2006). Surprisingly, previous exposure to *P. entomophila* produced the opposite effect to what we had expected in that it decreased acute‐phase resistance, which suggests that primary exposure came at a cost to resistance. The decrease in resistance was consistent across the four genotypes, although noticeably stronger for RAL765. A study on *T. castaneum* larvae primed against *Bacillus thuringiensis* showed that at 6 hrs post‐challenge primed beetles had lower bacterial loads compared to controls, but at 8 and 12 hrs post‐challenge, this pattern was reversed (Tate & Graham, 2017). Although *T. castaneum* were primed trans‐generationally rather than within generation, it shows that in theory priming can result in a transient increase in pathogen load in surviving primed animals (Tate & Graham, 2017).

### 4.2 Post‐challenge fecundity is genetically variable but is not influenced by primary exposure or challenge

Immune defences are costly and are assumed to be traded off against other life‐history traits such as reproduction. Likewise, immune priming could be costly, so that greater protection may come with greater costs to host fecundity. Despite these theoretical predictions, we observed no effect of previous exposure or challenge on fecundity; instead, it varied with genotype regardless of previous exposure or challenge. At least one empirical study has examined the costs of within‐generation immune priming (Contreras‐Garduño et al., 2014). Although there was no difference in the number of eggs that *Plasmodium berghei‐*primed and *Plasmodium berghei*‐non‐primed mosquitoes laid, eggs from primed females had lower hatching rates, indicating a reproductive cost to previous pathogen exposure (Contreras‐Garduño et al., 2014).

### 4.3 Fecundity tolerance is genotype‐dependent

We had reasoned that improved resistance in the individuals previously exposed to bacteria could come at a cost to tolerance if immune priming was mediated by a host resistance strategy. However, since we did not find evidence of improved resistance in the groups previously exposed to *L. lactis* or *P. entomophila*, and host fecundity remained relatively stable across treatment groups, we predicted a positive effect of primary exposure on tolerance, that is evidence for tolerance‐mediated immune priming (e.g., Tate, 2017) as recently observed for survival tolerance after oral priming and septic challenge with *Drosophila* C virus (Mondotte et al., 2018). However, we observed no such effect (Supporting Information Figure S4); rather, tolerance was determined by genotype, indicated by the significant interaction between bacterial load and genotype in response to infection with both bacteria species, and was likely driven by RAL765. The directionality of the tolerance reaction norms might be an indicator of genotype‐dependent expression of terminal investment: whereas RAL765 showed negative tolerance slopes, which suggests that the infection is costly with respect to fecundity, the other three genotypes showed more neutral or positive slopes, which might indicate terminal investment in reproduction. Genetic variation for tolerance is well established in animal systems (e.g., Blanchet, Rey, & Loot, 2010; Graham et al., 2011; Howick & Lazzaro, 2014; Parker, Garcia, & Gerardo, 2014; Råberg et al., 2007; Sternberg, Li, Wang, Gowler, & de Roode, 2013), but this is the first time that we have observed such variation for range tolerance in our infection system. This could be due to larger sample sizes than in a previous experiment, the fact that each genotype was selected specifically because they appeared to differ in disease tolerance and resistance, or other experimental design differences (Kutzer et al., 2018a).

### 4.4 Gene expression

In the snail *Biomphalaria glabrata*, antimicrobial peptides were significantly up‐regulated after priming and challenge with *Schistosoma* flatworms (Pinaud et al., 2016). Therefore, to test the hypothesis that the second stimulus (challenge) results in a stronger immune response when individuals have been previously exposed (Milutinović & Kurtz, 2016; Pradeu & Du Pasquier, 2018), we tested the strength of expression of two antimicrobial peptide genes. Contrary to our expectation, there was no primary exposure by challenge interaction, which would have indicated an increase in the strength of gene expression in those flies that had been previously exposed. Our results are consistent with those of Pham et al. (2007), who found that none of the three measured AMPs (*defensin, attacin* and *diptericin*) showed increased expression after priming and challenge with *Streptococcus pneumoniae*. There was a general increase in *Drs* and *Mtk* gene expression after infection with live *L. lactis*, where the degree to which it was up‐regulated depended upon the fly genotype. Interestingly, across genotypes, *Dscam1* showed a small but significant increase in gene expression after *L. lactis* challenge compared to a Ringer's challenge, a result that we did not observe after infecting *D. melanogaster* or *T. castaneum* with other bacterial species (Armitage et al., 2014; Peuß et al., 2016).

*Drs* expression was a good predictor of *Mtk* expression at the level of the individual, which would be predicted given that both genes are activated by the Toll pathway and induced by the same regulatory factor, deformed epidermal autoregulatory factor‐1 (DEAF‐1; Reed et al., 2008). The degree to which the immune system was activated seems to be independent of the bacterial load at 24 hrs (Supporting Information Figure S5); this is consistent with Duneau et al. (2017) who found no correlation between *Diptericin* expression and *Providencia rettgeri* load in *D. melanogaster* 8 hrs after infection.

### 4.5 Host genotype determines longer‐term survival after infection

We observed no effect of previous exposure on survival after challenge with either bacterial species, which suggests that the higher *P. entomophila* load at one DPC does not reflect either lower or higher survival in the longer term. Survival was determined by genotype, but not by the combination of genotype and primary exposure treatments: we had predicted the latter based on previous observations (Khan et al., 2016; Portela et al., 2013). The two fly genotypes showed different survival patterns after challenge with the two bacterial species: while *P. entomophila* induced almost 100% mortality in RAL379 5 days after challenge, <20% of RAL765 flies died in the same period. When one considers this in the light of our tolerance results, it appears that RAL765 invests more in survival at the cost of acute‐phase fecundity tolerance. This trend reversed after infection with *L. lactis*, whereby RAL765 had a higher mortality than RAL379. Of relevance here and above, cuticular piercing alone can lead to an increase in immune gene expression (e.g., Peuß et al., 2016), and we note that in the absence of a completely naïve treatment, we do not know the extent to which the piercing itself may have elicited a general elevation of the immune system and hence a general priming response.

### 4.6 In surviving flies, *L. lactis* forms a persistent infection but *P. entomophila* is cleared

*L. lactis* formed persistent chronic infections in survivors. Only one of 29 flies cleared the infection. Similarly, *D. melanogaster* infected with *P. rettgeri* had stable bacterial loads in the region of a few thousand bacteria until at least 10 days after infection, which Duneau et al. (2017) termed the set point bacterial load (SPBL). Our data extend the estimate for the duration of persistent bacterial infections to 28 days in *D. melanogaster,* and we suggest that we found evidence of a SPBL, given that the median bacterial load at one DPC was in the range of that found 28 days later. Previous exposure did not affect the bacterial load 28 DPC, but given the low number of survivors from which we could assay bacterial load, we may not have had the power to detect such an effect. *P. entomophila* was cleared from the haemocoel of surviving flies with the caveat that there were two RAL379 survivors. Duneau et al. (2017) observed that individuals infected with more benign bacterial species sometimes cleared the infection but in our case the survivors cleared the more pathogenic bacteria (note the different infection doses that we used for challenge with the two bacteria). We suggest that there may have been selection for qualitative resistance to *P. entomophila*, that is, on the survivors of these host genotypes to clear the infection.

## 5 CONCLUSIONS

Here we took a multi‐faceted approach to understand the role of phenotypic plasticity in insect immune defences, specifically whether fecundity, fecundity tolerance, and short‐ and longer‐term resistance and survival are affected by previous pathogen exposure. The phenotypic responses were, to some degree, dependent on host genotype, and the phenotypic responses to the two bacteria species contrasted in a number of ways, raising the question of why some bacteria persist but others do not. Our results illustrate the intricacies of immune defence phenotypes in an insect species.

## AUTHORS’ CONTRIBUTIONS

S.A.O.A. conceived the idea; J.K., M.A.M.K. and S.A.O.A. designed the experiments; M.A.M.K. & S.A.O.A. collected the data, analysed the data and led the writing of the manuscript. All authors contributed critically to the drafts and gave final approval for publication.

## Data Availability

Data available are from the Dryad Digital Repository: https://doi.org/10.5061/dryad.9kj41f0 (Kutzer, Kurtz, & Armitage, 2018b).

## References

[jane12953-bib-0501] Adamo, S. A. (1999). Evidence for adaptive changes in egg laying in crickets exposed to bacteria and parasites. Animal Behaviour, 57, 117–124. 10.1006/anbe.1998.0999 10053078

[jane12953-bib-0001] Armitage, S. A. O. , Sun, W. , You, X. , Kurtz, J. , Schmucker, D. , & Chen, W. (2014). Quantitative profiling of *Drosophila melanogaster* Dscam1 isoforms reveals no changes in splicing after bacterial exposure. PLoS One, 9, e108660 10.1371/journal.pone.0108660 25310676 PMC4195611

[jane12953-bib-0002] Armitage, S. A. O. , Thompson, J. J. W. , Rolff, J. , & Siva‐Jothy, M. T. (2003). Examining costs of induced and constitutive immune investment in *Tenebrio molitor* . Journal of Evolutionary Biology, 16, 1038–1044. 10.1046/j.1420-9101.2003.00551.x 14635919

[jane12953-bib-0003] Ayres, J. S. , & Schneider, D. S. (2008). A signaling protease required for melanization in *Drosophila* affects resistance and tolerance of infections. PLoS Biology, 6, 2764–2773. 19071960 10.1371/journal.pbio.0060305 PMC2596860

[jane12953-bib-0004] Bass, T. M. , Grandison, R. C. , Wong, R. , Martinez, P. , Partridge, L. , & Piper, M. D. W. (2007). Optimization of dietary restriction protocols in *Drosophila* . Journal of Gerontology, 62A, 1071–1081. 10.1093/gerona/62.10.1071 PMC4335187 17921418

[jane12953-bib-0005] Best, A. , Tidbury, H. , White, A. , & Boots, M. (2013). The evolutionary dynamics of within‐generation immune priming in invertebrate hosts. Journal of the Royal Society Interface, 10, 20120887. 10.1098/rsif.2012.0887 PMC3565738 23269850

[jane12953-bib-0006] Best, A. , White, A. , & Boots, M. (2008). Maintenance of host variation in tolerance to pathogens and parasites. Proceedings of the National Academy of Sciences of the United States of America, 105, 20786–20791. 10.1073/pnas.0809558105 19088200 PMC2634923

[jane12953-bib-0007] Blanchet, S. , Rey, O. , & Loot, G. (2010). Evidence for host variation in parasite tolerance in a wild fish population. Evolutionary Ecology, 24, 1129–1139. 10.1007/s10682-010-9353-x

[jane12953-bib-0008] Boman, H. G. , Nilsson, I. , & Rasmuson, B. (1972). Inducible antibacterial defence system in *Drosophila* . Nature, 237, 232–235. 10.1038/237232a0 4625204

[jane12953-bib-0009] Boots, M. , & Begon, M. (1993). Trade‐offs with resistance to a granulosis virus in the indian meal moth, examined by a laboratory evolution experiment. Functional Ecology, 7, 528–534. 10.2307/2390128

[jane12953-bib-0502] Contreras‐Garduño, J. , Rodríguez, M. C. , Rodríguez, M. H. , Alvarado‐Delgado, A. , & Lanz‐Mendoza, H. , (2014). Cost of immune priming within generations: trade‐off between infection and reproduction. Microbes and Infection, 16(3), 261–267, 10.1016/j.micinf.2013.11.010 24291714

[jane12953-bib-0503] Contreras‐Garduño, J. , Lanz‐Mendoza, H. , Franco, B. , Nava, A. , Pedraza‐Reyes, M. , & Canales‐Lazcano, J. (2016). Insect immune priming: ecology and experimental evidence. Ecological Entomology, 41, 351–366. 10.1111/een.12300

[jane12953-bib-0011] Dhinaut, J. , Chogne, M. , & Moret, Y. (2018). Immune priming specificity within and across generations reveals the range of pathogens affecting evolution of immunity in an insect. Journal of Animal Ecology, 87, 448–463. 10.1111/1365-2656.12661 28239855

[jane12953-bib-0012] Duneau, D. , Ferdy, J. B. , Revah, J. , Kondolf, H. , Ortiz, G. A. , Lazzaro, B. P. , & Buchon, N. (2017). Stochastic variation in the initial phase of bacterial infection predicts the probability of survival in *D. melanogaster* . ELife, 6, e28298 10.7554/eLife.28298 29022878 PMC5703640

[jane12953-bib-0013] Futo, M. , Sell, M. P. , Kutzer, M. A. M. , & Kurtz, J. (2017). Specificity of oral immune priming in the red flour beetle *Tribolium castaneum* . Biology Letters, 13, 20170632 10.1098/rsbl.2017.0632 29237813 PMC5746539

[jane12953-bib-0014] Gandon, S. , Mackinnon, M. J. , Nee, S. , & Read, A. F. (2001). Imperfect vaccines and the evolution of pathogen virulence. Nature, 414, 751–756. 10.1038/414751a 11742400

[jane12953-bib-0015] González‐Tokman, D. M. , González‐Santoyo, I. , Lanz‐Mendoza, H. , & Córdoba Aguilar, A. (2010). Territorial damselflies do not show immunological priming in the wild. Physiological Entomology, 35, 364–372. 10.1111/j.1365-3032.2010.00752.x

[jane12953-bib-0016] Graham, A. L. , Shuker, D. M. , Pollitt, L. C. , Auld, S. K. J. R. , Wilson, A. J. , & Little, T. J. (2011). Fitness consequences of immune responses: Strengthening the empirical framework for ecoimmunology. Functional Ecology, 25, 5–17. 10.1111/j.1365-2435.2010.01777.x

[jane12953-bib-0017] Haine, E. R. , Moret, Y. , Siva‐Jothy, M. T. , & Rolff, J. (2008). Antimicrobial defense and persistent infection in insects. Science, 322, 1257–1259. 10.1126/science.1165265 19023083

[jane12953-bib-0018] Howick, V. M. , & Lazzaro, B. P. (2014). Genotype and diet shape resistance and tolerance across distinct phases of bacterial infection. BMC Evolutionary Biology, 14, 56 10.1186/1471-2148-14-56 24655914 PMC3997931

[jane12953-bib-0019] Jackson, J. A. , Hall, A. J. , Friberg, I. M. , Ralli, C. , Lowe, A. , Zawadzka, M. , … Begon, M. (2014). An immunological marker of tolerance to infection in wild rodents. PLoS Biology, 12, 566–578. 10.1371/journal.pbio.1001901 PMC4086718 25004450

[jane12953-bib-0020] Khan, I. , Agashe, D. , & Rolff, J. (2017). Early‐life inflammation, immune response and ageing. Proceedings of the Royal Society B: Biological Sciences, 284, 20170125 10.1098/rspb.2017.0125 PMC5360934 28275145

[jane12953-bib-0021] Khan, I. , Prakash, A. , & Agashe, D. (2016). Divergent immune priming responses across flour beetle life stages and populations. Ecology and Evolution, 6, 7847–7855. 10.1002/ece3.2532 30128134 PMC6093166

[jane12953-bib-0022] Kraaijeveld, A. R. , & Godfray, H. C. J. (1997). Trade‐off between parasitoid resistance and larval competitive ability in *Drosophila melanogaster* . Nature, 389, 278–280. 10.1038/38483 9305840

[jane12953-bib-0023] Kurtz, J. , & Franz, K. (2003). Evidence for memory in invertebrate immunity. Nature, 425, 37–38. 10.1038/425037a 12955131

[jane12953-bib-0024] Kutzer, M. A. M. , & Armitage, S. A. O. (2016a). Maximising fitness in the face of parasites: A review of host tolerance. Zoology, 119, 281–289. 10.1016/j.zool.2016.05.011 27373338

[jane12953-bib-0025] Kutzer, M. A. M. , & Armitage, S. A. O. (2016b). The effect of diet and time after bacterial infection on fecundity, resistance, and tolerance in *Drosophila melanogaster* . Ecology and Evolution, 6, 4229–4242. 10.1002/ece3.2185 27386071 PMC4884575

[jane12953-bib-0026] Kutzer, M. A. M. , Kurtz, J. , & Armitage, S. A. O. (2018a). Genotype and diet affect resistance, survival, and fecundity but not fecundity tolerance. Journal of Evolutionary Biology, 31, 159–171. 10.1111/jeb.13211 29150962

[jane12953-bib-0027] Kutzer, M. A. M. , Kurtz, J. , & Armitage, S. A. O. (2018b). Data from: A multi‐faceted approach testing the effects of previous bacterial exposure on resistance and tolerance. *Dryad Digital Repository*, 10.5061/dryad.9kj41f0 PMC6487967 30697699

[jane12953-bib-0028] Lazzaro, B. P. (2002). A population and quantitative genetic analysis of the Drosophila melanogaster antibacterial immune response (PhD Thesis). The Pennsylvania State University, State College, Pennsylvania.

[jane12953-bib-0029] Lazzaro, B. P. , Sackton, T. B. , & Clark, A. G. (2006). Genetic variation in *Drosophila melanogaster* resistance to infection: A comparison across bacteria. Genetics, 174, 1539–1554. 10.1534/genetics.105.054593 16888344 PMC1667071

[jane12953-bib-0504] Leulier, F. , Rodriguez, A. , Khush, R. S. , Abrams, J. M. , & Lemaitre, B. (2000). The Drosophila caspase Dredd is required to resist gram‐negative bacterial infection. EMBO Reports, 1(4), 353–358. 10.1093/embo-reports/kvd073 11269502 PMC1083747

[jane12953-bib-0505] Levashina, E. A. , Ohresser, S. , Bulet, P. , Reichhart, J. M. , Hetru, C. , & Hoffmann, J. A. (1995). Metchnikowin, a novel immune‐inducible proline‐rich peptide from Drosophila with antibacterial and antifungal properties. European Journal of Biochemistry, 233(2), 694–700. 10.1111/j.1432-1033.1995.694_2.x 7588819

[jane12953-bib-0506] Linder, J. E. , Owers, K. A. , & Promislow, D. E. L. (2008). The effects of temperature on host‐pathogen interactions in D. melanogaster: who benefits? Journal of Insect Physiology, 54(1): 297–308. 10.1016/j.jinsphys.2007.10.001 17981291 PMC3390751

[jane12953-bib-0030] Little, T. J. , & Kraaijeveld, A. R. (2004). Ecological and evolutionary implications of immunological priming in invertebrates. Trends in Ecology and Evolution, 19, 58–60. 10.1016/j.tree.2003.11.011 16701227

[jane12953-bib-0031] Little, T. J. , Shuker, D. M. , Colegrave, N. , Day, T. , & Graham, A. L. (2010). The coevolution of virulence: Tolerance in perspective. PLoS Pathogens, 6, e1001006 10.1371/journal.ppat.1001006 20838464 PMC2936544

[jane12953-bib-0032] Longdon, B. , Cao, C. , Martinez, J. , & Jiggins, F. M. (2013). Previous exposure to an RNA virus does not protect against subsequent infection in *Drosophila melanogaster* . PLoS One, 8, e73833 10.1371/journal.pone.0073833 24040086 PMC3770682

[jane12953-bib-0033] Mackay, T. F. C. , Richards, S. , Stone, E. A. , Barbadilla, A. , Ayroles, J. F. , Zhu, D. , … Gibbs, R. A. (2012). The *Drosophila melanogaster* Genetic Reference Panel. Nature, 482, 173–178. 10.1038/nature10811 22318601 PMC3683990

[jane12953-bib-0034] Milutinović, B. , & Kurtz, J. (2016). Immune memory in invertebrates. Seminars in Immunology, 28, 328–342. 10.1016/j.smim.2016.05.004 27402055

[jane12953-bib-0035] Mondotte, J. A. , Gausson, V. , Frangeul, L. , Blanc, H. , Lambrechts, L. , & Saleh, M.‐C. (2018). Immune priming and clearance of orally acquired RNA viruses in *Drosophila* . Nature Microbiology, 3, 1394–1403. 10.1038/s41564-018-0265-9 30374170

[jane12953-bib-0036] Moret, Y. , & Siva‐Jothy, M. T. (2003). Adaptive innate immunity? Responsive‐mode prophylaxis in the mealworm beetle, *Tenebrio molitor* . Proceedings of the Royal Society of London, Series B: Biological Sciences, 270, 2475–2480. 10.1098/rspb.2003.2511 14667338 PMC1691523

[jane12953-bib-0037] Neyen, C. , Poidevin, M. , Roussel, A. , & Lemaitre, B. (2012). Tissue‐ and ligand‐specific sensing of gram‐negative infection in drosophila by PGRP‐LC isoforms and PGRP‐LE. Journal of Immunology, 189, 1886–1897. 10.4049/jimmunol.1201022 22772451

[jane12953-bib-0038] Parker, B. J. , Garcia, J. R. , & Gerardo, N. M. (2014). Genetic variation in resistance and fecundity tolerance in a natural host‐pathogen interaction. Evolution, 68, 2421–2429. 24689981 10.1111/evo.12418

[jane12953-bib-0039] Peuß, R. , Wensing, K. U. , Woestmann, L. , Eggert, H. , Milutinović, B. , Sroka, M. G. U. , … Armitage, S. A. O. (2016). Down syndrome cell adhesion molecule 1: Testing for a role in insect immunity, behaviour and reproduction. Royal Society Open Science, 3, 00–00. 10.1098/rsos.160138 PMC4852650 27152227

[jane12953-bib-0040] Pham, L. N. , Dionne, M. S. , Shirasu‐Hiza, M. , & Schneider, D. S. (2007). A specific primed immune response in *Drosophila* is dependent on phagocytes. PLoS Pathogens, 3, e26 10.1371/journal.ppat.0030026 17352533 PMC1817657

[jane12953-bib-0041] Pinaud, S. , Portela, J. , Duval, D. , Nowacki, F. C. , Olive, M. A. , Allienne, J. F. , … Gourbal, B. (2016). A shift from cellular to humoral responses contributes to innate immune memory in the vector snail *Biomphalaria glabrata* . PLoS Pathogens, 12, e1005361 10.1371/journal.ppat.1005361 26735307 PMC4703209

[jane12953-bib-0042] Portela, J. , Duval, D. , Rognon, A. , Galinier, R. , Boissier, J. , Coustau, C. , … Gourbal, B. (2013). Evidence for specific genotype‐dependent immune priming in the lophotrochozoan *Biomphalaria glabrata* snail. Journal of Innate Immunity, 5, 261–276. 10.1159/000345909 23343530 PMC6741461

[jane12953-bib-0043] Pradeu, T. , & Du Pasquier, L. (2018). Immunological memory: What's in a name? Immunological Reviews, 283, 7–20. 10.1111/imr.12652 29664563

[jane12953-bib-0044] R Core Team . (2016). R: A language and environment for statistical computing. Vienna, Austria: R Foundation for Statistical Computing Retrieved from https://www.R-project.org/

[jane12953-bib-0045] Råberg, L. , Graham, A. L. , & Read, A. F. (2009). Decomposing health: Tolerance and resistance to parasites in animals. Philosophical Transactions of the Royal Society of London. Series B, Biological Sciences, 364, 37–49. 18926971 10.1098/rstb.2008.0184 PMC2666700

[jane12953-bib-0046] Råberg, L. , Sim, D. , & Read, A. F. (2007). Disentangling genetic variation for resistance and tolerance to infectious diseases in animals. Science, 318, 812–814. 10.1126/science.1148526 17975068

[jane12953-bib-0047] Read, A. F. , Baigent, S. J. , Powers, C. , Kgosana, L. B. , Blackwell, L. , Smith, L. P. , … Nair, V. K. (2015). Imperfect vaccination can enhance the transmission of highly virulent pathogens. PLoS Biology, 13, e1002198 10.1371/journal.pbio.1002198 26214839 PMC4516275

[jane12953-bib-0048] Reber, A. , & Chapuisat, M. (2012). No evidence for immune priming in ants exposed to a fungal pathogen. PLoS One, 7, e35372 10.1371/journal.pone.0035372 22523588 PMC3327680

[jane12953-bib-0507] Reed, D. E. , Huang, X. M. , Wohlschlegel, J. A. , Levine, M. S. , & Senger, K. (2008). DEAF‐1 regulates immunity gene expression in Drosophila. Proceedings of the National Academy of Sciences of the United States of America, 105(24), 8351–8356. 10.1073/pnas.0802921105 18550807 PMC2448840

[jane12953-bib-0049] Rodrigues, J. , Brayner, F. A. , Alves, L. C. , Dixit, R. , & Barillas‐Mury, C. (2010). Hemocyte differentiation mediates innate immune memory in *Anopheles gambiae* mosquitoes. Science, 329, 1353–1355. 10.1126/science.1190689 20829487 PMC3510677

[jane12953-bib-0050] Roth, O. , Sadd, B. M. , Schmid‐Hempel, P. , & Kurtz, J. (2009). Strain‐specific priming of resistance in the red flour beetle, *Tribolium castaneum* . Proceedings of the Royal Society of London. Series B, Biological Sciences, 276, 145–151. 10.1098/rspb.2008.1157 18796392 PMC2614262

[jane12953-bib-0051] Roy, B. A. , & Kirchner, J. W. (2000). Evolutionary dynamics of pathogen resistance and tolerance. Evolution, 54, 51–63. 10.1111/j.0014-3820.2000.tb00007.x 10937183

[jane12953-bib-0052] Sadd, B. M. , & Schmid‐Hempel, P. (2006). Insect immunity shows specificity in protection upon secondary pathogen exposure. Current Biology, 16, 1206–1210. 10.1016/j.cub.2006.04.047 16782011

[jane12953-bib-0053] Sadd, B. M. , & Siva‐Jothy, M. T. (2006). Self‐harm caused by an insect's innate immunity. Proceedings of the Royal Society of London. Series B, Biological Sciences, 273, 2571–2574. 10.1098/rspb.2006.3574 16959651 PMC1634900

[jane12953-bib-0054] Sternberg, E. D. , Lefèvre, T. , Li, J. , de Castillejo, C. L. F. , Li, H. , Hunter, M. D. , & de Roode, J. C. (2012). Food plant derived disease tolerance and resistance in a natural butterfly‐plant‐parasite interactions. Evolution, 66, 3367–3376. 10.1111/j.1558-5646.2012.01693.x 23106703

[jane12953-bib-0055] Sternberg, E. D. , Li, H. , Wang, R. , Gowler, C. , & de Roode, J. C. (2013). Patterns of host‐parasite adaptation in three populations of monarch butterflies infected with a naturally occurring protozoan disease: Virulence, resistance, and tolerance. The American Naturalist, 182, E235–E248. 10.1086/673442 24231547

[jane12953-bib-0056] Tate, A. T. (2017). A general model for the influence of immune priming on disease prevalence. Oikos, 126, 350–360. 10.1111/oik.03274

[jane12953-bib-0057] Tate, A. T. , & Graham, A. L. (2017). Dissecting the contributions of time and microbe density to variation in immune gene expression. Proceedings of the Royal Society of London. Series B, Biological Sciences, 284, 20170727 10.1098/rspb.2017.0727 28747473 PMC5543217

[jane12953-bib-0058] Tate, A. T. , & Rudolf, V. H. W. (2012). Impact of life stage specific immune priming on invertebrate disease dynamics. Oikos, 121, 1083–1092. 10.1111/j.1600-0706.2011.19725.x

[jane12953-bib-0059] Vodovar, N. , Vinals, M. , Liehl, P. , Basset, A. , Degrouard, J. , Spellman, P. , … Lemaitre, B. (2005). *Drosophila* host defense after oral infection by an entomopathogenic *Pseudomonas* species. Proceedings of the National Academy of Sciences of the United States of America, 102, 11414–11419. 10.1073/pnas.0502240102 16061818 PMC1183552

[jane12953-bib-0060] Watson, F. L. , Püttmann‐Holgado, R. , Thomas, F. , Lamar, D. L. , Hughes, M. , Kondo, M. , … Schmucker, D. (2005). Extensive diversity of Ig‐superfamily proteins in the immune system of insects. Science, 309, 1874–1878. 16109846 10.1126/science.1116887

[jane12953-bib-0061] Werner, T. , Liu, G. , Kang, D. , Ekengren, S. , Steiner, H. , & Hultmark, D. (2000). A family of peptidoglycan recognition proteins in the fruit fly *Drosophila melanogaster* . Proceedings of the National Academy of Sciences of the United States of America, 97, 13772–13777. 11106397 10.1073/pnas.97.25.13772 PMC17651

[jane12953-bib-0062] Wickham, H. (2009). ggplot2: Elegant graphic for data analysis. New York, NY: Springer, 566–210.

