# Supplementary material for: A multi‐faceted approach testing the effects of previous bacterial exposure on resistance and tolerance
Source: J Anim Ecol. 2019 Mar 6;88(4):566–78. doi: 10.1111/1365-2656.12953 (PMC6487967; doi:10.1111/1365-2656.12953)
Supplement: Supplementary file 1 [file JANE-88-566-s001.pdf]

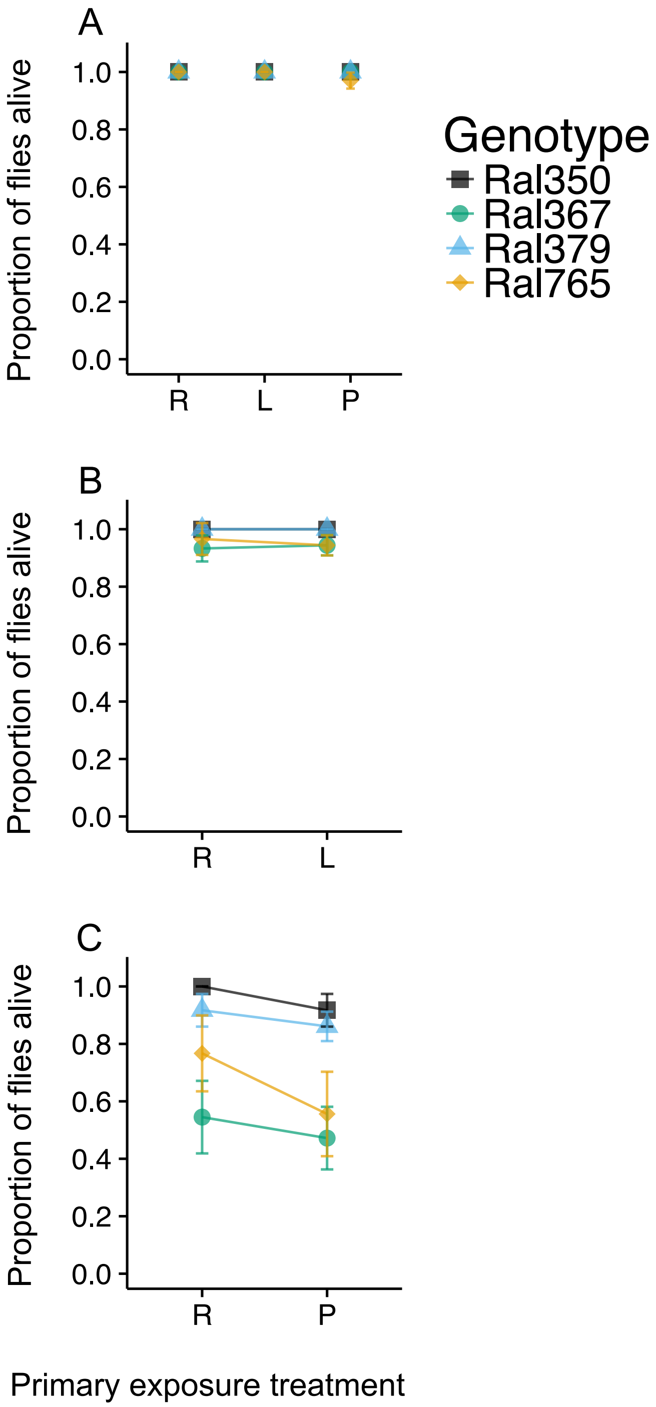

Fig. S1. Survival one DPC. The proportion of flies alive one day post challenge is shown on the y-axis and primary exposure−challenge treatment combinations are shown on the x-axis, where R: Ringer's, L: *L. lactis* and P: *Pseudomonas entomophila*. The legend indicates the four fly genotypes. (**A**) Females first injected with R, heat-killed L or P and seven days later challenged with R. (**B**) Females first injected with R or L and then challenged with live L. (**C**) Females first injected with R or P and challenged with live P. Lines represents the survival reaction norm of one of the four genotypes in each primary exposure-challenge group, each data point is the mean survival from 6 replicate experiments. Error bars represent one standard error. For statistics see Table S2.
