# Supplementary material for: A multi‐faceted approach testing the effects of previous bacterial exposure on resistance and tolerance
Source: J Anim Ecol. 2019 Mar 6;88(4):566–78. doi: 10.1111/1365-2656.12953 (PMC6487967; doi:10.1111/1365-2656.12953)
Supplement: Supplementary file 2 [file JANE-88-566-s002.pdf]

Overall correlation, n = 8
S = 38, p = 0.17, Rho = 0.55
T = 19, p = 0.28, tau = 0.36

Overall correlation, n = 8
S = 16, p = **0.02**, Rho = 0.81
T = 23, p = **0.03**, tau = 0.64

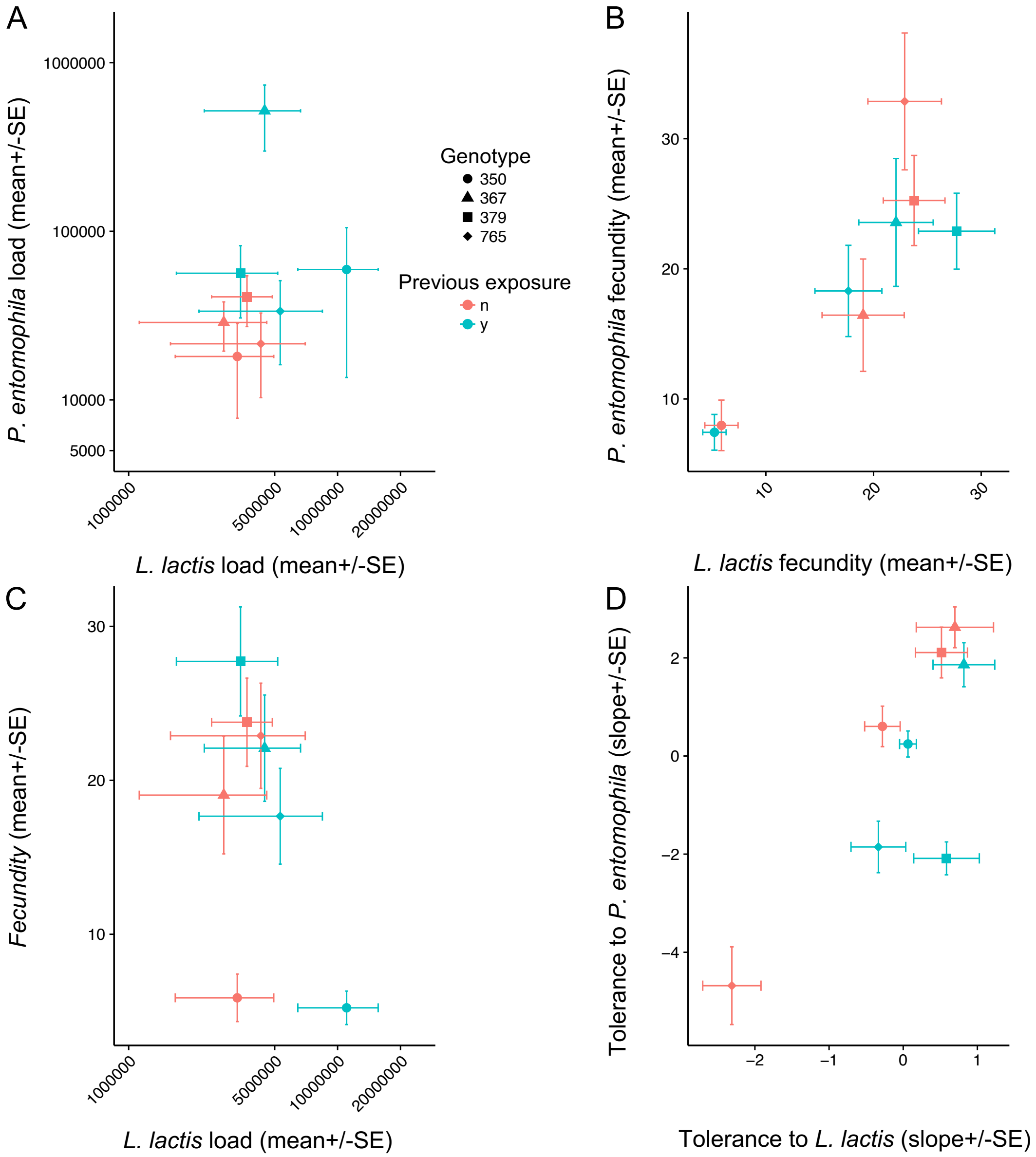

Overall correlation, n = 8
S = 110, p = 0.4618, Rho = -0.31
T = 9, p = 0.2751, tau -0.36
Previous exposure, n = 4
S = 20, p = 0.08, Rho = -1
T = 0, p = 0.08, tau = -1
Ringer's, n = 4
S = 4, p = 0.43, Rho = 0.6
T = 4, p = 0.75, tau = 0.33

Overall correlation, n = 8
S = 30, p-value = 0.096, Rho = 0.64
T = 21, p-value = 0.11, tau 0.5
Previous exposure, n = 4
S = 6, p = 0.75, Rho = 0.4
T = 4, p = 0.75, tau = 0.33
Ringer's, n = 4
S = 0, p = 0.08, Rho = 1
T = 6, p = 0.08, tau = 1

Fig. S2. Correlations between bacteria load (resistance), fecundity and tolerance in Experiment 1. (**A**) Relationship between *P. entomophila* load and *L. lactis* load one day post live challenge. (**B**) Relationship between *L. lactis* load and fecundity one day post live challenge. (**C**) Significant positive correlation between fecundity from *P. entomophila* and *L. lactis* infected females one day post infection. (**D**) Relationship between tolerance to *P. entomophila* and tolerance to *L. lactis*. The legend illustrates the genotypes and the primary exposure treatment. Statistical values for Spearman's Rho and Kendall's tau as well as the corresponding p-values are above or below each panel.
