# Supplementary material for: A multi‐faceted approach testing the effects of previous bacterial exposure on resistance and tolerance
Source: J Anim Ecol. 2019 Mar 6;88(4):566–78. doi: 10.1111/1365-2656.12953 (PMC6487967; doi:10.1111/1365-2656.12953)
Supplement: Supplementary file 3 [file JANE-88-566-s003.pdf]

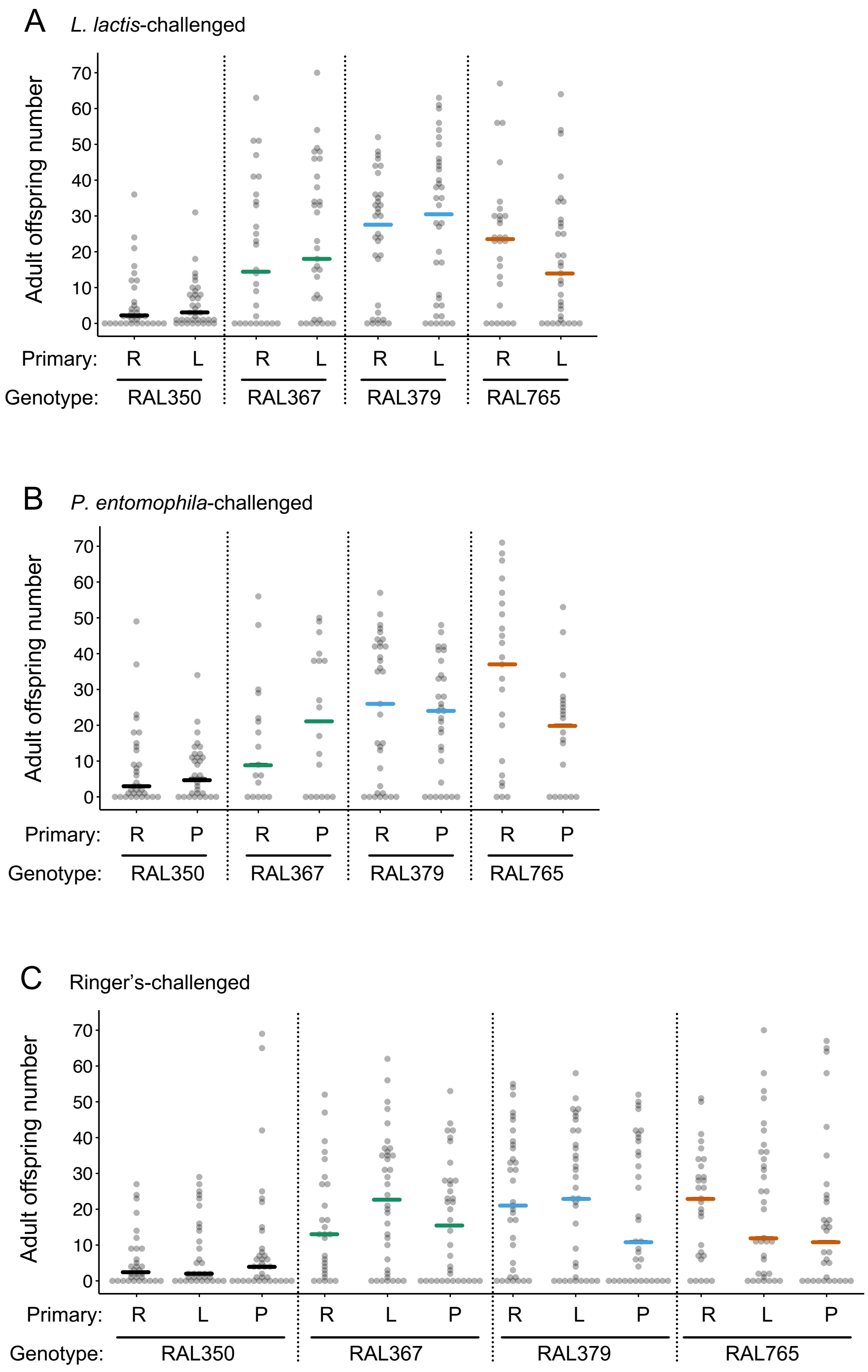

Fig. S3. Fecundity one DPC. Fecundity on the y-axis was measured as the number of adult offspring produced. The x-axis shows fly genotypes and the primary exposure treatments, where R: Ringer's, L: *L. lactis* and P: *P. entomophila*. Seven days after primary exposure flies were challenged with either (**A**) *L.* lactis, (**B**) *P. entomophila* or (**C**) Ringer's. Each dot is the number of adult offspring produced by one fly in the 24 hours after challenge. The coloured lines show median fecundity. For statistics see Table S3.
