# Supplementary material for: A multi‐faceted approach testing the effects of previous bacterial exposure on resistance and tolerance
Source: J Anim Ecol. 2019 Mar 6;88(4):566–78. doi: 10.1111/1365-2656.12953 (PMC6487967; doi:10.1111/1365-2656.12953)
Supplement: Supplementary file 4 [file JANE-88-566-s004.pdf]

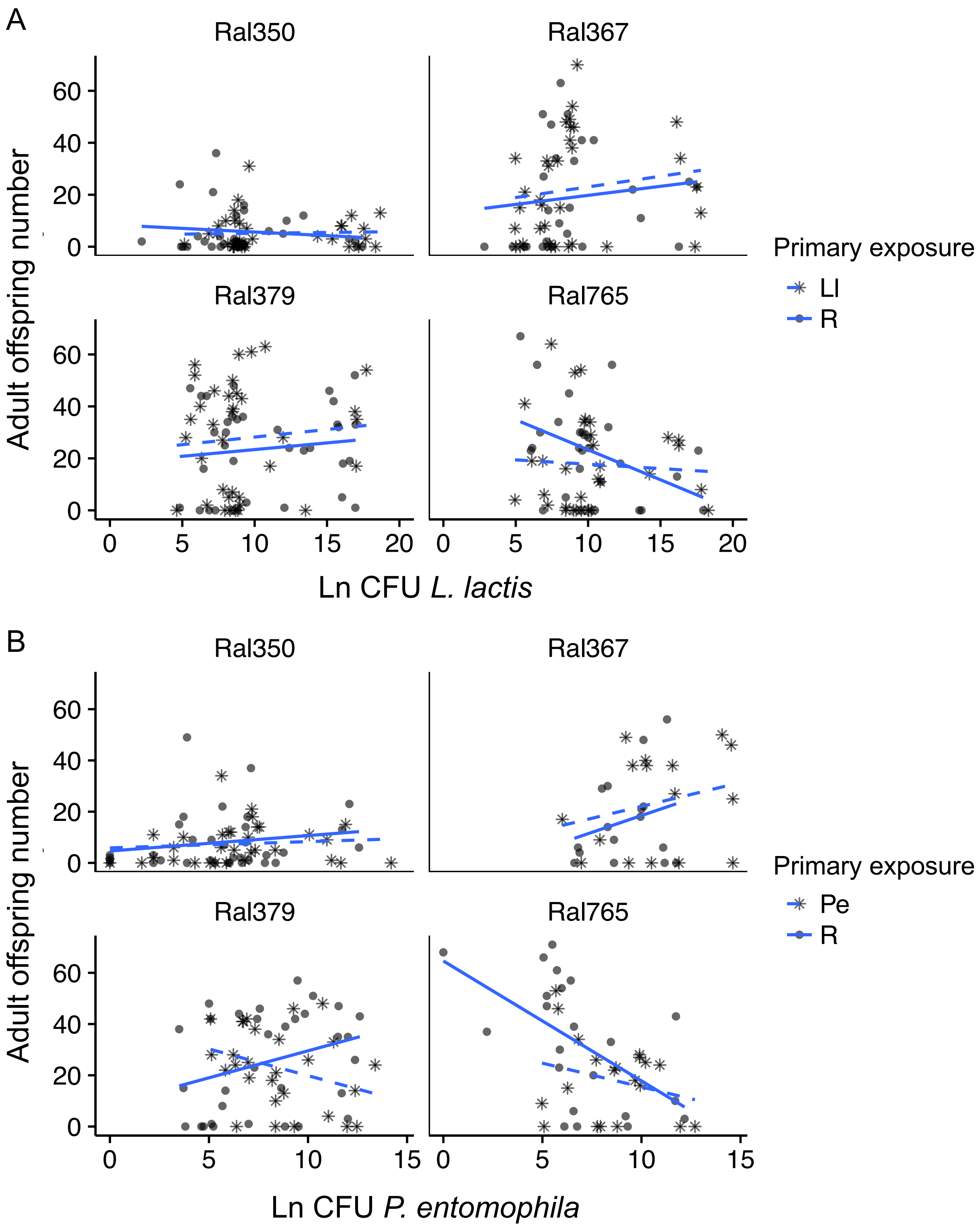

Fig. S4. Fecundity tolerance in relation to primary exposure and genotype 24 hours post challenge. Tolerance curves are plotted for each genotype and primary exposure-challenge treatment combination. (**A**) shows flies challenged with *L. lactis* and (**B**) shows flies challenged with *P. entomophila.*
