# Supplementary material for: A multi‐faceted approach testing the effects of previous bacterial exposure on resistance and tolerance
Source: J Anim Ecol. 2019 Mar 6;88(4):566–78. doi: 10.1111/1365-2656.12953 (PMC6487967; doi:10.1111/1365-2656.12953)
Supplement: Supplementary file 5 [file JANE-88-566-s005.pdf]

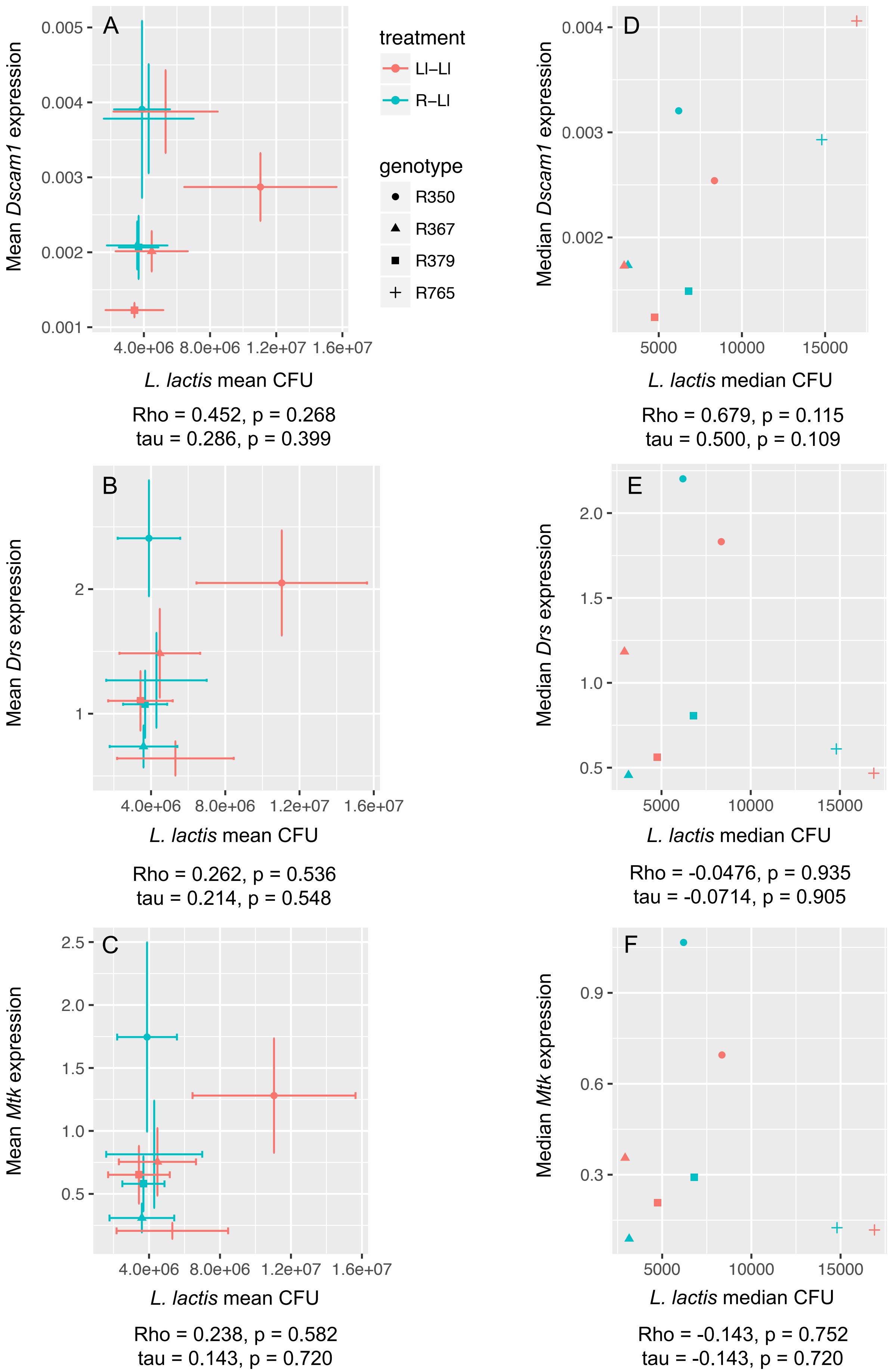

Fig. S5. Correlations between gene expression and *L. lactis* load. (**A**)-(**C**) Correlations between mean gene expression and mean *L. lactis* CFU count where each data point results from the mean and standard error from different individuals from the same treatment group / genotype. (**D**)-(**F**) Correlations between median gene expression and median CFU count per treatment group / genotype. The legend illustrates the primary exposure-challenge treatment where R is Ringer's and Ll is *L. lactis,* and the symbols indicate the genotypes. Statistical values for Spearman's Rho and Kendall's tau as well as the corresponding p-values are below each panel.
