# Supplementary material for: A multi‐faceted approach testing the effects of previous bacterial exposure on resistance and tolerance
Source: J Anim Ecol. 2019 Mar 6;88(4):566–78. doi: 10.1111/1365-2656.12953 (PMC6487967; doi:10.1111/1365-2656.12953)
Supplement: Supplementary file 6 [file JANE-88-566-s006.docx]

**Table S1**. **Primers for qPCR***.* qPCR efficiencies (E). An arrow within primer sequences indicates where an intron lies within a primer. *Drs* and *Mtk* each have only one exon.

| *Gene symbol* | *Name* | *Flybase ID* | *Target or reference gene* | *E / Fragment lenth (bp)* | *5’ – 3’ primer sequence* | *Primer origin* |
| --- | --- | --- | --- | --- | --- | --- |
| *Drs* | *Drosomycin* | FBgn0010381 | Target | 1.945 / 79 | F: CTGCCTGTCCGGAAGATACAA  R: TCCCTCCTCCTTGCACACA | Ref. 1 |
| *Dscam1* | *Down syndrome cell adhesion molecule 1* | Fbgn0033159 | Target | 2.000 / 105 | F: TAAGGCCTTCGCCCAGGGATCC  R: TCTCCGGGGGTGTCGC↓CAACT | Ref. 2 |
| *Mtk* | *Metchnikowin* | FBgn0014865 | Target | 1.939 / 98 | F: GCTACATCAGTGCTGGCAGA  R: ATTGGACCCGGTCTTGGTTG | F: Ref. 3  R: This study |
| *rp49* | *Ribosomal protein 49/L32* | FBgn0002626 | Reference | 2.000 / 97 | F: CGCCCAGCATACAGGCCCAA  R: TGCGCCATTTGTG↓CGACAGC | Ref. 4 |
| *rpL13a* | *Ribosomal protein L13a* | FBgn0037351 | Reference | 1.980 / 104 | F: AAGGCAGTCCGAG↓GCATGATCCC  R: CGACGCTTGTCGTAGGGCGA | Ref. 2 |

References

1. Fellous, S. & Lazzaro, B.P. (2011) Potential for evolutionary coupling and decoupling of larval and adult immune gene expression. *Molecular Ecology.* **20**, 1558-1567.
2. Armitage, S.A.O., Sun, W., You, X., Kurtz, J., Schmucker, D. & Chen, W. (2014) Quantitative profiling of Drosophila melanogaster Dscam1 isoforms reveals no changes in splicing after bacterial exposure. *PLoS ONE*, **9**, e108660.
3. Tsai, C.W., McGraw, E.A., Ammar, E.-D., Dietzgen, R.G. & Hogenhout, S.A. (2008) *Drosophila melanogaster* mounts a unique immune response to the rhabdovirus *Sigma virus*. *Applied and Environmental Micobiology,* **74**, 3251-3256.
4. Peuß, R., Wensing, K.U., Woestmann, L., Eggert, H., Milutinović, B., Sroka, M.G.U., Scharsack, J.P., Kurtz, J. & Armitage, S.A.O. (2016) Down syndrome cell adhesion molecule 1: Testing for a role in insect immunity, behaviour and reproduction. *Royal Society Open Science*, **3**.
