# Supplementary material for: A multi‐faceted approach testing the effects of previous bacterial exposure on resistance and tolerance
Source: J Anim Ecol. 2019 Mar 6;88(4):566–78. doi: 10.1111/1365-2656.12953 (PMC6487967; doi:10.1111/1365-2656.12953)
Supplement: Supplementary file 7 [file JANE-88-566-s007.docx]

**Table S2. Survival one DPC.** The effect of genotype and primary exposure treatment (Ringer’s (R), *L. lactis* (L), or *P. entomophila* (P)) on fly survival 24 hours after injection with either Ringer’s solution (R), *L. lactis* (L), or *P. entomophila* (P). The first of the two letters (R, L or P) denotes the primary exposure treatment where bacteria were heat-killed and the second of the two letters is the challenge treatment where live bacteria were injected.

|  | *Tested effect* | *Df* | *Dev* | *Resid.df* | *Resid.dev* | *P* |  |
| --- | --- | --- | --- | --- | --- | --- | --- |
| Model 1a: | Genotype | 3 | 10.51 | 44 | 19.14 | **0.015** |  |
| R-L vs L-L | Primary | 1 | 0.01 | 43 | 19.12 | 0.9 |  |
|  | Genotype x Primary | 3 | 0.19 | 40 | 18.94 | 0.97 |  |
|  |  | *Df* | *Dev* | *Resid.df* | *Resid.dev* | *F* | *P* |
| Model 1b: | Genotype | 3 | 52.78 | 22 | 87.72 | 10.05 | **<0.0001** |
| R-P vs P-P | Primary | 1 | 4.95 | 43 | 82.88 | 2.82 | 0.1 |
|  | Genotype x Primary | 3 | 3.39 | 40 | 79.39 | 0.65 | 0.59 |
