# Supplementary material for: A multi‐faceted approach testing the effects of previous bacterial exposure on resistance and tolerance
Source: J Anim Ecol. 2019 Mar 6;88(4):566–78. doi: 10.1111/1365-2656.12953 (PMC6487967; doi:10.1111/1365-2656.12953)
Supplement: Supplementary file 8 [file JANE-88-566-s008.docx]

**Table S3. Fecundity one DPC**. The effect of genotype, primary exposure and challenge on post-infection fecundity. Pre-infection fecundity was included as a covariate. The models are split by the bacteria used for primary exposure and challenge. Model 3a tests the effect of genotype and *L. lactis* primary exposure and challenge on post-challenge fecundity compared to Ringer’s injected controls. Model 3b tests the effect of genotype and *P. entomophila* primary exposure and challenge on post-challenge fecundity in comparison to Ringer’s injected controls.

|  | Model 3a: *L. lactis* | | | Model 3b: *P. entomophila* | | | |
| --- | --- | --- | --- | --- | --- | --- | --- |
| *Tested effect* | *χ^2^* | *Df* | *P* | *χ^2^* | *Df* | *P* |  |
| Genotype | 53.97 | 3 | **<0.0001** | 20.01 | 3 | **0.00017** |  |
| Primary | 0.30 | 1 | 0.58 | 0.44 | 1 | 0.51 |  |
| Challenge | 0.20 | 1 | 0.65 | 0.07 | 1 | 0.79 |  |
| Genotype x Primary | 2.72 | 3 | 0.43 | 4.20 | 3 | 0.24 |  |
| Genotype x Challenge | 0.77 | 3 | 0.85 | 2.25 | 3 | 0.52 |  |
| Primary x Challenge | 1.31 | 1 | 0.25 | 2.53 | 1 | 0.11 |  |
| Genotype x Primary x Challenge | 0.76 | 3 | 0.86 | 3.93 | 3 | 0.27 |  |
| Pre-infection fecundity | 304.68 | 1 | **<0.0001** | 223.97 | 1 | **<0.0001** |  |
