# Supplementary material for: A multi‐faceted approach testing the effects of previous bacterial exposure on resistance and tolerance
Source: J Anim Ecol. 2019 Mar 6;88(4):566–78. doi: 10.1111/1365-2656.12953 (PMC6487967; doi:10.1111/1365-2656.12953)
Supplement: Supplementary file 9 [file JANE-88-566-s009.docx]

**Appendix S1**

Supplementary methods

*Mating assay*

Approximately 18 hours before mating, individual males were placed into vials supplemented with live yeast (Experiment 1: Replicates 1–4 = 90 males per genotype; Replicates 5 & 6 = 120 males per genotype; Experiment 2: 220 per genotype, per replicate). The following morning, individual females were transferred to a vial containing a male of the same genotype. We recorded the time the female entered the vial. Copulations were observed, and the start and finish time recorded. Every fly was given a maximum of two and a half hours to mate. Any flies mating for less than five minutes were discarded, as this likely means that sperm were not transferred (Gilchrist & Partridge 2000). Males were removed from each vial following copulation and discarded to prevent remating.

*RNA extraction and DNase treatment*

After homogenising the flies with a pestle over liquid nitrogen, we added 500 μl of TriFast^TM^ and the samples were incubated for ~15 mins at room temperature and vortexed two times during incubation. The samples were centrifuged at room temperature, 15,871 rcf for 10 minutes. Thereafter the supernatant was transferred to a new tube and mixed with 100 μl chloroform and incubated for 2-3 minutes with shaking in between. The samples were centrifuged at 4 ºC, 15,871 rcf for 15 minutes. The upper aqueous phase was transferred to a new tube and 250 μl isopropanol was added. The samples were incubated for one hour at -20 ºC then centrifuged at 4 ºC, 15,871 rcf for 15 minutes. After removing the supernatant, the pellet was washed with 1000 μl of 70 % ethanol, the ethanol was removed, and the pellet was allowed to dry at 37 ºC for 20 minutes. The resulting RNA pellet was resuspended in 15 μl water and subsequently frozen at -80 ºC. Before a DNase treatment, the RNA was diluted 1:1 with water. Thereafter we used the DNA-free^TM^ kit (Ambion) according to the manufacturer’s instructions. The RNA concentration and quality were measured using an Implen NanoPhotometer. We performed two reverse transcription reactions, one with oligodT primers and one with random hexamer primers. For the reverse transcription of the RNA, SuperScript III^TM^ (Invitrogen) was used according to the manufacturer’s instructions, with oligodT or random hexamer primers and 500 ng of RNA for each reaction in a total volume of 10 μl. The cDNA produced with oligodT primers was used for qPCR analyses using two target genes (*Drosomycin* and *Metchnikowin*; Table S1) and two reference genes (*rpL13a* and *rp49;* Table S1). The cDNA produced with random hexamer primers was used for qPCR analyses using *Down syndrome cell adhesion molecule 1* (*Dscam1*; Table S1) as a target gene and *rpL13a* as a reference gene. Amplification efficiencies (E) of the primer pairs were determined with five dilutions (undiluted, 1:10, 1:100, 1:1,000, 1:10,000) of template cDNA, where E = 10^-1/slope^. The qPCRs were performed in a 384-well plate format, with a total reaction volume of 10 μl in each well. Two technical replicate qPCR reactions were performed per cDNA sample. Each reaction contained 0.5 μl cDNA, 4.9 μl of water, 4 μl Kapa SYBR® Fast qPCR Mastermix, and 0.3 μl of each of the forward and reverse primers at a concentration of 5 pmol/μl. The reactions were run on a LightCycler480 (Roche) using the following protocol: 95 °C for 3 min, followed by 40 cycles of annealing and amplification at 60 °C for one min and denaturation at 95ºC for 10 sec. As a final step the products were heated up to 97 °C with continuous fluorescence measurements to obtain the melting curves and subsequently cooled to 40 °C. All plates contained non-template controls for all primer pairs, where the cDNA was replaced with water. The crossing thresholds (C_T_) values were obtained using the second derivative maximum method, and the mean C_T_ of the technical replicates was used for statistical analyses.

*Statistical analyses*

We used the nlme (Pinheiro *et al.* 2015), MASS (Ripley *et al.* 2015), lme4 (Bates *et al.* 2015), car (Fox & Weisberg 2002), survival (Therneau & Grambsch 2000; Therneau 2015a), and coxme (Therneau 2015b) packages in our statistical analyses. Model specifics are listed below for clarity. We tested for main effects and interactions using Wald F and *χ^2^* tests in the car package (Bolker *et al.* 2009). Model reduction did not result in significantly better fits (assessed with AIC values) and did not change the interpretation of our results, therefore we chose to use the full models.

*Exp. 1: Effect of previous exposure and genotype on survival one DPC*

We tested for changes in survival due to primary exposure treatment (Primary) and genotype using generalized linear models with binomial (1a) or quasibinomial (1b) error structures (glm function, nlme package). Each experimental replicate was treated as a separate data point for every genotype x priming combination, so we excluded the random effect of replicate in these analyses. Model 1a compared survival between R−L and L−L to test for an effect of previous exposure to *L. lactis*. Model 1b compared survival between R−P and P−P to test for an effect of previous exposure to *P. entomophila*. There were no differences in survival among the control groups (R−R, L−R, P−R) so we do not include this model.

Model 1a *_L. lactis_*, b *_P. entomophila_*: Survival ~ Genotype x Primary

*Exp. 1: Effect of previous exposure and genotype on bacteria load one DPC*

We sub-divided the dataset by bacteria challenge species and used bacteria load measured as colony forming units (CFU) as our response variable to model host resistance (the inverse of bacteria load) to a live bacterial challenge following an initial exposure to heat killed bacteria. Model 2a tested the effect of previous exposure to heat-killed *L. lactis* by comparing the R−L and L−L treatments and Model 2b tested the effect of previous exposure to heat killed *P. entomophila* by comparing the R−P and P−P treatments. Both response variables (*L. lactis* CFU and *P. entomophila* CFU) were natural log transformed and conformed to the assumptions of normality, therefore we used linear mixed effects models (lmer function, lme4 package). The models are detailed below.

Model 2a, b: log(CFU) ~ Genotype * Primary + (1|Replicate/ Block)

*Exp. 1: Effect of previous exposure, challenge and genotype on post challenge fecundity*

We tested for the effects of previous exposure, challenge and genotype on post-challenge fecundity. Model 3a compared fecundity among R−R, R−L, L−R and L−L treatment groups and Model 3b compared fecundity among the R−R, R−P, P−R and P−P treatment groups. We did not include the flies infected with *L. lactis* that we used for RTqPCR in our fecundity analysis. We used generalized linear mixed models with poisson errors (glmer function, lme4 package) and included pre-infection fecundity as a covariate, although the results were similar with or without the covariate. The addition of pre-infection fecundity resulted in a better model fit. FlyID accounted for overdispersion.

Model 3a, b: Post-challenge fecundity ~ Pre-challenge fecundity + Genotype * Primary * Challenge + (1|Replicate) + (1|FlyID)

*Exp. 1: Effect of previous exposure and genotype on host tolerance to infection*

We used post challenge fecundity as our response variable to examine the effects of previous bacterial exposure on tolerance to challenge with *L. lactis* or *P. entomophila*. We used natural log transformed bacteria load in these models to normalize the distribution of the residuals. Model 4a evaluated the effects of bacteria load, genotype and previous exposure on tolerance to *L. lactis* and Model 4b evaluated the effects of bacteria load, genotype and previous exposure on tolerance to *P. entomophila* (glmer function, lme4 package). We included FlyID as an individual level random effect in both models to account for overdispersion. A significant interaction with bacteria load and any of the experimental factors would indicate that there is variation for host tolerance.

Model 4a: Post-challenge fecundity *_L. lactis_* ~ CFU * Genotype * Primary + (1|Replicate) + (1|FlyID)

Model 4b: Post-challenge fecundity *_P. entomophila_* ~ CFU * Genotype * Primary + (1|Replicate) + (1|FlyID)

We also tested 12 correlations between mean traits (mean per genotype / primary exposure treatment), and present the results of those which showed trends (p < 0.1) or in one case was statistically significant (Figure S2). The 12 correlations tested were: 1) *P. entomophila* load 1 dpc vs *L. lactis* load 1 dpc; 2) fecundity in *L. lactis* challenged flies vs. *L. lactis* load 1 dpc; 3) fecundity in *P. entomophila* challenged flies vs *P. entomophila* load 1 dpc; 4) *P. entomophila* post challenge fecundity vs. *L. lactis* post challenge fecundity; 5) *L. lactis* survival 1 dpc vs *L. lactis* load 1 dpc; 6) *P. entomophila* survival 1 dpc vs *P. entomophila* load 1 dpc; 7) *L. lactis* survival 1 dpc vs *L. lactis* fecundity 1 dpc; 8) *P. entomophila* survival 1 dpc vs *P. entomophila* fecundity 1 dpc; 9) tolerance to *L. lactis* 1 dpc vs resistance to *L. lactis* 1 dpc; 10) tolerance to *P. entomophila* 1 dpc vs resistance to *P. entomophila* 1 dpc; 11) tolerance to *L. lactis* 1 dpc vs tolerance to *P. entomophila* 1 dpc; 12) tolerance to *P. entomophila* 1 dpc vs *P. entomophila* survival 1 dpc.

*Exp. 1: Effect of previous exposure and genotype on gene expression of* L. lactis *infected flies*

We tested for the effects of genotype, primary exposure treatment (R or L) and challenge (R or L) on relative gene expression using linear mixed effects models. To obtain relative target gene expression values for each sample, we applied the formula 2^-ΔCT^ (Schmittgen & Livak 2008), where ΔC_T_ = (C_T_ target gene expression - C_T_ geometric mean of reference gene expression). Our aim was to analyse the effects of more than one factor, i.e. we were interested in genotype, previous exposure and challenge (whilst controlling for the random effect of replicate and block) on gene expression. Therefore we calculated 2^-ΔCt^ and used this as our response variable. This formula does not take the efficiency of the qPCR into account. Rough guidelines for the comparative CT method (2^-ΔΔCt^), which also does not take the efficiency of the qPCR into account, suggest that if the efficiency of the reference and target genes are within 10 % of each other then this is similar enough to be able to use the CT method (Schmittgen & Livak 2008). Given that in our case the differences in efficiencies of the target genes compared to their respective reference gene(s) were all below this 10 % rough guideline: *Drosomycin*: 4.8 %, *Dscam1*: 1 %, *Metchnikowin*: 5.4 %, we reasoned that it would also be within the acceptable range for the 2^-ΔCt^ method. The response for each gene, i.e. *Dscam1, Drosomycin* and *Metchnikowin*, was natural log transformed and tested in a separate model.

Model 5a-c: log(Relative gene expression) ~ Genotype * Primary * Challenge + (1|Replicate/Block)

We tested whether expression of the three genes was correlated across individuals with the following models.

Model 5d-f: log(Relative gene expression) ~ log(Relative gene expression) + (1|Replicate/Block)

To account for testing multiple genes and the increased likelihood of type I errors, we corrected the p-values with the Benjamini and Hochberg (1995) method in R using p.adjust and the false discovery rate (“fdr”). We calculated adjusted p-values for each of the independent variables and their interactions (see Koppik & Fricke 2017).

*Exp. 2: Effect of previous exposure and genotype on survival for 28 DPC*

Fly survival over the 28 days following the challenge was analysed using day of death as the response variable and flies that were still alive at the end of the experiment were included as censored cases. We split the data into subsets according to challenge treatment and ran three separate models. Model 6a compared survival between Ringer’s controls, i.e., L-R, P-R and R−R, Model 6b compared survival between R−L and L−L to test for an effect of previous exposure to *L. lactis* and Model 6c compared survival between R−P and P−P to test for an effect of previous exposure to *P. entomophila*.

Model 6a, b, c: Day of death ~ Genotype * Primary + (1|Replicate/ Block)

All models were additionally tested without the random effects by using Cox proportional hazards. Furthermore, we tested whether the Cox regression models fulfilled the assumptions of proportional hazards over time using cox.zph (Grambsch & Therneau 1994). When the models did not fulfill the assumptions we also used the survival regression (“survreg”) function. In all cases the coxme, coxph models and survreg models gave qualitatively similar results.

A number of studies have followed survival after priming until 1 to 2 weeks post-challenge and found effects of priming on survival. We wondered if there might be an early survival advantage to RAL379 of being primed with *L. lactis*, and whether this was later masked by aging/general vigour. Therefore, we also tested whether there is an effect of previous exposure to survival until day nine for the *L. lactis* subset of data. However, this was not the case: Priming x Genotype: Chi square = 1.35, p = 0.24; Genotype: Chi square = 0.014, p = 0.91; Priming: Chi square = 0.83, p = 0.36.

*Exp. 2: Effect of previous exposure and genotype on bacterial load 28 DPC*

Only *L. lactis-*injected flies contained CFUs 28 DPC. We tested the effect of primary exposure (R or L) and genotype (Ral765 or Ral379) on natural log transformed *L. lactis* load (CFU count) with linear mixed effects models. The full model was:

Model 7: log(CFU) ~ Genotype * Primary + (1|Replicate/ Block)

**References**

Bates, D., Maechler, M., Bolker, B. & Walker, S. (2015) Fitting Linear Mixed-Effects Models Using lme4. *Journal of Statistical Software*, **67**, 1–48.

Benjamini, Y., & Hochberg, Y. (1995). Controlling the false discovery rate—A

practical and powerful approach to multiple testing. *Journal of the Royal Statistical Society. Series B (Methodological)*, **57**, 289–300.

Bolker, B.M., Brooks, M.E., Clark, C.J., Geange, S.W., Poulsen, J.R., Stevens, M.H.H. & White, J.-S.S. (2009) Generalized linear mixed models: a practical guide for ecology and evolution. *Trends in Ecology & Evolution*, **24**, 127–135.

Fox, J. & Weisberg, S. (2002) An {R} Companion to Applied Regression. *Sage Publications*, 2–3.

Grambsch, P.M. & Therneau, T.M. (1994) Proportional hazards tests and diagnostics based on weighted residuals. *Biometrika*, **81**, 515–526.

Koppik, M. & Fricke, C. (2017) Gene expression changes in male accessory glands during ageing are accompanied by reproductive decline in *Drosophila melanogaster*. *Molecular Ecology*, **26**, 6704–6716.

Pinheiro, J., Bates, D., DebRoy, S., Sarkar, D. & R Core Team. (2015) Linear and Nonlinear Mixed Effects Models.

Ripley, B., Venables, B., Bates, D., Hornik, K., Gebhardt, A. & Firth, D. (2015) Support Functions and Datasets for Venables and Ripley’s MASS.

Therneau, T.M. (2015a) A Package for Survival Analysis in S. *Survival*.

Therneau, T.M. (2015b) coxme: Mixed Effects Cox Models.

Therneau, T.M. & Grambsch, P.M. (2000) Modeling Survival Data: Extending the Cox Model.
